# Evaluation of the Anti-Amyloid and Anti-Inflammatory Properties of a Novel Vanadium(IV)–Curcumin Complex in Lipopolysaccharides-Stimulated Primary Rat Neuron-Microglia Mixed Cultures

**DOI:** 10.3390/ijms26010282

**Published:** 2024-12-31

**Authors:** Georgios Katsipis, Sophia N. Lavrentiadou, George D. Geromichalos, Maria P. Tsantarliotou, Eleftherios Halevas, George Litsardakis, Anastasia A. Pantazaki

**Affiliations:** 1 Laboratory of Biochemistry, Department of Chemistry, Aristotle University of Thessaloniki, 54124 Thessaloniki, Greece; gkatsipis@chem.auth.gr (G.K.); lefterishalevas@gmail.com (E.H.); 2 Center for Interdisciplinary Research and Innovation, Laboratory of Neurodegenerative Diseases (LND), Thermi, 57001 Thessaloniki, Greece; gerom@chem.auth.gr; 3 Laboratory of Animal Physiology, School of Veterinary Medicine, Aristotle University of Thessaloniki, 54124 Thessaloniki, Greece; mtsant@vet.auth.gr; 4 Laboratory of Inorganic Chemistry, Department of Chemistry, Aristotle University of Thessaloniki, 54124 Thessaloniki, Greece; 5 Institute of Biosciences & Applications, National Centre for Scientific Research “Demokritos”, 15310 Athens, Greece; 6 Laboratory of Materials for Electrotechnics, School of Electrical and Computer Engineering, Aristotle University of Thessaloniki, 54124 Thessaloniki, Greece; lits@eng.auth.gr

**Keywords:** curcumin, vanadium-curcumin complex, mixed neuron-glia cultures, neuroinflammation, amyloid precursor protein, lipopolysaccharides

## Abstract

Lipopolysaccharides (LPS) are bacterial mediators of neuroinflammation that have been detected in close association with pathological protein aggregations of Alzheimer’s disease. LPS induce the release of cytokines by microglia and mediate the upregulation of inducible nitric oxide synthase (iNOS)—a mechanism also associated with amyloidosis. Curcumin is a recognized natural medicine but has extremely low bioavailability. V-Cur, a novel hemocompatible Vanadium(IV)-curcumin complex with higher solubility and bioactivity than curcumin, is studied here. Co-cultures consisting of rat primary neurons and microglia were treated with LPS and/or curcumin or V-Cur. V-Cur disrupted LPS-induced overexpression of amyloid precursor protein (APP) and the in vitro aggregation of human insulin (HI), more effectively than curcumin. Cell stimulation with LPS also increased full-length, inactive, and total iNOS levels, and the inflammation markers IL-1β and TNF-α. Both curcumin and V-Cur alleviated these effects, with V-Cur reducing iNOS levels more than curcumin. Complementary insights into possible bioactivity mechanisms of both curcumin and V-Cur were provided by In silico molecular docking calculations on Aβ_1-42_, APP, Aβ fibrils, HI, and iNOS. This study renders curcumin-based compounds a promising anti-inflammatory intervention that may be proven a strong tool in the effort to mitigate neurodegenerative disease pathology and neuroinflammatory conditions.

## 1. Introduction

More than a century after the initial description of Alzheimer’s disease (AD), its exact pathophysiological etiology is still unknown, and no successful treatment has been introduced [1]. AD is characterized by the gradual build-up of amyloid beta (Aβ) senile plaques, and neurofibrillary tangles comprised of hyper-phosphorylated microtubule-associated tau protein. Other pathological hallmarks of AD include extensive astrogliosis and microgliosis (neuroinflammation), oxidative stress and neurodegeneration, which ultimately lead to cerebral atrophy and dementia [2].

Aβ peptides are generated by the successive proteolytic processing of the amyloid precursor protein (APP). The 42-amino acid Aβ peptide (Aβ_1-42_) in particular, is recognized as the main component of senile plaques, due to its increased tendency for aggregation [3]. The “amyloid cascade hypothesis”, namely the theory that Aβ aggregation is the central cause of the pathological pathways implicated in AD, is the most prevalent theory for the pathogenesis of the disease [4]. However, anti-amyloid therapies have been ineffective in managing AD pathology [5]. This observation implies that there may be a crucial physiological role of APP and its proteolytic products, with the anti-microbial nature of Aβ_1-42_ gaining continuously increasing attention in the latest decade [6,7].

Microglia are the most prominent immune cells of the brain, comprising about 10% of the brain cells. In addition to their significant role in the development of the central nervous system (CNS), microglia are also implicated in the alleviation of injury lesions, and the phagocytosis of microbes and abnormal protein aggregates, like those of Aβ. Microglia are crucial in the response of CNS to LPS through recognition from the toll-like receptor 4 (TLR4), which they highly express [8]. Triggering of TLR4 leads to the expression of proinflammatory cytokines, such as IL-1β and TNF-α, and production of free radicals, including but not limited to reactive oxygen species (ROS). In addition, upregulation of the inducible NO synthase (iNOS) has been documented and contributes to the oxidative response of microglia, due to the release of nitrate radicals [9]. Though imperative for fighting off invading pathogens, overactivated microglia may cause toxic effects on neurons, which may culminate in neurodegeneration [10].

Curcumin (C_21_H_20_O_6_) is a polyphenol isolated from the turmeric plant (*Curcuma longa*), and has been long studied for its various therapeutic potentials as a natural, cost-effective alternative to usual interventions [11,12], including neuroprotective and anti-neurotoxic effects [13]. Curcumin inhibits Aβ aggregation, promotes clearance of senile plaques and hinders neuroinflammation and oxidative stress, and is thus suggested as a promising candidate for the prevention of AD [14]. However, the medicinal exploitation of curcumin is hindered by its extremely low bioavailability and low water solubility [11]. Serum levels of curcumin hardly reach therapeutic titers [15] and the distribution of curcumin in the brain is also limited [16], even though it can cross the blood–brain barrier (BBB) [17].

Various nano-formulations, including liposomes [18,19] and nanoparticles [20,21], have been previously designed to increase the ability of curcumin to cross the BBB, its uptake by neuronal cells, or its antioxidant and anti-inflammatory potential. However, these syntheses come with the drawbacks of high cost, instability in water suspensions and incompatibility with oral consumption [22,23]. Another approach includes chemical modification and metal complexation, which would increase its water solubility, stability and retention in the blood and the brain parenchyma, without the need for sophisticated and expensive transfer systems [11,24]. Though metal-curcumin complexes have proven beneficial in chronic inflammatory diseases [25], up to date only few such studies have been performed in cells or in vivo, involving Curcumin–Cu^2+^, -Zn^2+^ [26], -Fe^3+^, and -Mn^2+^ complexes [27].

Vanadium(IV)—curcumin complex (V-Cur) is a fully characterized analog of curcumin with increased solubility and antioxidant capacity [28] and non-toxic to eukaryotic cells (yeast and erythrocytes) in concentrations up to 15 μΜ [28,29]. V-Cur has the potential of reversible, higher binding affinity for blood serum albumin and DNA compared to curcumin [28,29,30]. In addition, V-Cur has been efficiently encapsulated in magnetic liposomes, which gradually release curcumin, enhance its solubility and bioavailability, and allow magnetism-assisted targeting/monitoring, thus providing a new perspective and for future in vivo applications [31]

In the current study, the potential anti-inflammatory and anti-amyloidogenic effects of a V-Cur have been investigated in mixed co-cultures of primary neurons and microglia, isolated from neonatal rat brain. Western blotting was used to study the effect of LPS, V-Cur and curcumin treatment on the protein levels of APP, active and inactivated iNOS, and the inflammatory markers TNF-α and IL-1β. A fibrillization study with human insulin (HI) and In silico molecular docking studies were also employed to offer new insights into the observed effects of curcumin and V-Cur on the crucial amyloid pathway associated with AD pathology. The results shed light on the effects of LPS on neuronal cells and demonstrate the beneficial effects of curcumin or its modified form on neuroinflammatory diseases.

## 2. Results

### 2.1. APP Levels in Mixed Neuron-Microglia Cultures Stimulated with LPS

**Figure 1:**
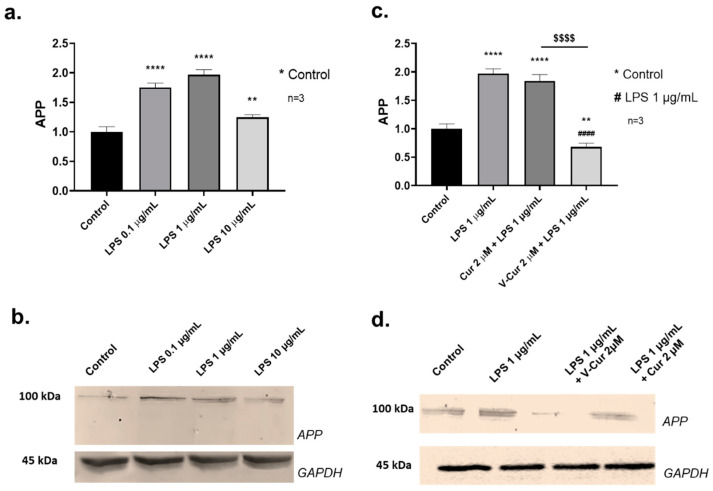
Levels of amyloid precursor protein (APP) in mixed cultures of primary neuron-microglia, in the absence or presence of 0.1, 1, or 10 μg/mL of LPS (**a**,**b**). Effect of LPS (1 μg/mL) in the presence or absence of 2 μΜ curcumin or V-Cur complex on APP levels (**c**,**d**). Analysis performed with Western blotting. Glyceraldehyde-3-phosphate dehydrogenase (GAPDH) was used to verify equal loading. The density of the blots was semi-quantified with ImageJ 1.54. Results are presented as fractional changes in comparison with the control sample and are the mean (±SD) of three independent biological experiments. One-way ANOVA was employed to compare untreated or LPS-treated samples. Statistical significance when compared with: * untreated sample (control); # LPS-treated sample; $ curcumin-treated: * *p* < 0.05; ** *p* < 0.01; *** *p* < 0.001; **** *p* < 0.0001; #### *p* < 0.0001; $$$$ *p* < 0.0001.

To investigate the ability of the rat neuron-microglia co-culture system to mimic the brain pathology, the co-cultures were exposed to LPS, and their response was evaluated by means of APP induction. The co-cultures were treated with 0.1, 1 and 10 μg/mL of LPS for 24 h and APP levels in cell lysates were determined by Western blotting (Figure 1a,b). All concentrations of LPS tested were effective in significantly increasing APP levels, with the maximum response mediated by 1 μg/mL of LPS (1.97-fold increase compared to that of the control cells, *p* < 0.0001). Therefore, in all further experiments, LPS were administered to the cells at the dose of 1 μg/mL.

### 2.2. The Complex V-Cur Effectively Inhibits the LPS-Induced Increase of APP Levels

To determine the protective effect of curcumin, the co-cultures were pre-treated with 2 μM curcumin or 2 μM V-Cur 30 min prior to the addition of 1 μg/mL LPS. Cells were harvested and lysed after 24 h, and APP levels were determined by SDS-PAGE and immunoblotting (Figure 1c,d). As demonstrated in Figure 1c, V-Cur but not curcumin, inhibited the induction of APP by LPS. Cultures that were pre-treated with curcumin presented a 1.74-fold increase in APP levels, compared to a 1.97-fold change mediated by LPS alone (*p* > 0.05). On the other hand, cultures that were treated with V-Cur presented decreased APP levels compared to the control samples (0.68-fold change, *p* = 0.0024) or the LPS-treated samples (*p* < 0.0001).

### 2.3. Molecular Docking Calculations on Aβ_1-42_, Aβ Fibril and APP

Due to the anti-amyloid properties of curcumin [32], and to further study the effect of curcumin on APP and amyloid peptides, pure curcumin as well as V-Cur were also evaluated In silico for their binding affinity with APP, Aβ, and Aβ fibrils, to further assess the potential to employ these two compounds in AD management. As explained in detail in the following sections, In silico studies indicated that both compounds exhibit a particularly good binding affinity for APP, Aβ_1-42_ monomer and Aβ fibrils. In addition, V-Cur exhibited higher binding affinity in comparison with curcumin, as deduced by the predicted binding energies (Table 1). More details on the interactions of curcumin or V-Cur with APP, Aβ_1-42_ peptide and Aβ fibrils are also provided in the Supplementary Materials.

#### 2.3.1. Amyloid β Precursor Protein (APP)

**Figure 2:**
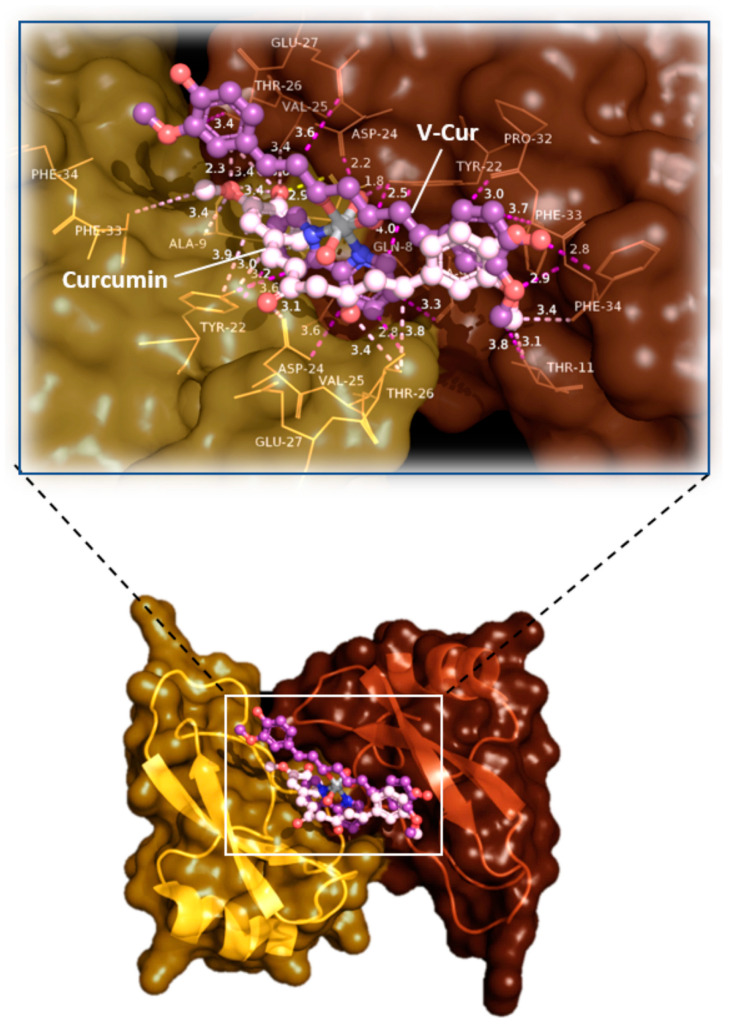
Docking poses orientation of curcumin and V-Cur in the crystal structure of the Kunitz protease inhibitor domain (APPI) of APP (PDB accession number 1AAP). The target protein is illustrated as a semi-transparent cartoon and surface colored in yellow orange and chocolate (chains A and B, respectively), while curcumin and V-Cur molecules are rendered in ball-and-stick mode and colored according to atom type in light pink and violet purple C atoms, respectively. The ligand binding site of both molecules depicting the architecture of the binding interactions is also illustrated (in the upper part) with an additional depiction of selected contacting amino acid residues of the binding pocket rendered in line and colored according to the cartoon. Binding interactions are illustrated in light pink (for curcumin) and violet (for V-Cur). Heteroatom color code: V: grey, N: blue, and O: red. Molecular docking simulations of both ligands were performed individually. Hydrogen atoms are omitted for clarity. The final structure was ray-traced and illustrated with the aid of PyMol Molecular Graphics.

To evaluate binding interactions between curcumin and V-Cur with APP, molecular docking studies were adopted with the employment of the Kunitz protease inhibitor domain (APPI) of APP, consisting of a twisted two-stranded antiparallel beta-sheet followed by an alpha helix [33]. Both curcumin and V-Cur have shown a particularly good binding affinity for APPI, with V-Cur exhibiting lower binding energy compared to curcumin, hence suggesting a better binding capacity with APPI (Table 1). The docking procedure reveals that curcumin and V-Cur lie within the best binding pocket and interact with nearby residues by H-bond, polar, π-polar, π-anion, π-cation, π-alkyl, and hydrophobic interactions. Further stabilization of the docked molecules inside the proteolysis targeting site is achieved with the formation of π-π stacking interactions between the aromatic rings of curcumin and V-Cur and Y22 of both chains A and B (Figure 2 and Supplementary Table S1). These computational studies offer a new insight in the biochemical results from mixed neuron-glia cultures, suggesting that, in addition to impairing APP upregulation under inflammatory processes, curcumin and V-Cur hopefully can also assist in impeding the proteolytic processing of APP to toxic Aβ fragments. Further analysis of the binding interactions of curcumin and V-Cur on APPI is reported in Supplementary Materials.

#### 2.3.2. Aβ_1-42_ Monomer

Aβ_1-42_ peptide shows two helical stretches connected by a kink region centered on a regular type I β-turn involving the residues 25–28 (G25, S26, N27, and K28). The molecular docking of either curcumin or V-Cur on Aβ_1-42_ is depicted in Supplementary Figure S5. Notably, the docking procedure reveals that the Aβ central hydrophobic core recognition sequence KLVFFAED (Aβ_16-23_) could be the target of both curcumin and V-Cur. From the binding affinity values summarized in Table 1, it is deduced that V-Cur exhibits higher binding affinity for A*β*_1-42_ monomer compared to curcumin. It is thus hypothesized that higher negative binding energy would provide stronger ligand binding and, thus, slower oligomerization process. This was also demonstrated by Viet et al. by determining the relationship between binding affinity and aggregation rate [34].

Molecular docking studies suggest that both curcumin and V-Cur interact with Aβ_1-42_ through hydrogen bonding (H-bonds) as well as by side-chain interactions. This serves in destabilized conformation states of the peptide and hence disruption of fibril propagation. This seems to be attributed specifically to perturbation of the intramolecular H-bonds between H13, K16, L17, V18, F19, F20, A21, E22, D23, V24, G25, S26, N27, K28, G29, A30, I31, I32, G33, and L34, at the KLVFFAED sequence, as well as in the turn position of the peptide—the part of the hinge region in contact with the C-terminal region (possibly disrupting the “bend” segment between the two helices). This immediate effect on H-bonding imposed by both curcumin and V-Cur, probably leads to structural transformation of the Aβ_1-42_ peptides, leading to the destabilization of their secondary structure. Molecular Dynamics simulation studies could be employed in the future to further verify these disrupting interactions.

#### 2.3.3. Aβ Fibril

The model for Aβ fibril used in the study consists of residues 17–42 of the Aβ_1-42_ (L17–A42) composing a beta-strand-turn-beta-strand motif, that contains two intermolecular, parallel, in-register beta-sheets formed by residues 17–26 (L17–S26) (beta1) and 31–42 (I31–A42) (beta2) in a pentamer (chains A–E) construction. Both curcumin and V-Cur are predicted to bind strongly to the Aβ fibril and inhibit further elongation processes. Also, docking studies suggest that both compounds can act as β-sheet-disrupters of Aβ (17–42) fibril formation. In Supplementary Figure S6, curcumin and V-Cur are illustrated to be inserted in the edge of the β-sheets assembly (fibrillar ends), in a perpendicular to fibril axis manner, making contacts with residues of all five chains of Aβ fibrils. As two β-sheets constitute a parallel β-sandwich that is stabilized by inter-β-sheet side-chain contacts in Aβ fibrils, curcumin and V-Cur plausibly seem to disrupt this stabilization by interrupting the neighboring inter-β-sheet contacts.

### 2.4. Curcumin and V-Cur Inhibit the Formation of Human Insulin Amyloids (Insulin Fibrillation)

**Figure 3:**
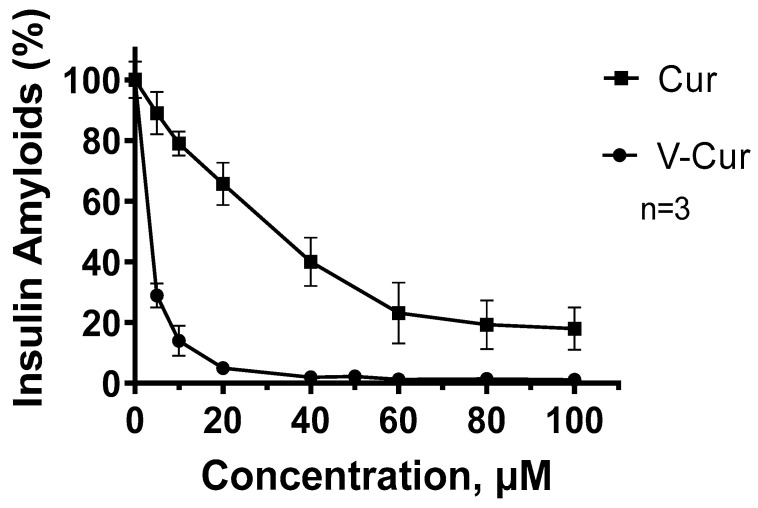
In vitro fibrillation assay with insulin in the presence of several concentrations (0–100 μΜ) of either curcumin (■) or V-Cur (●). The insulin amyloid fibers formed in the absence or presence of either curcumin or V-Cur were semi-quantified by employing Thioflavin T fluorescence, with excitation at 450 nm and recording the emission spectrum at 490 nm. The results from three independent experiments are provided as mean normalized fibrillization rates (±SEM), setting the value of the control sample as 100%. Some error bars are not visible due to very small values (<1%).

To further explore the potency of curcumin or its complex V-Cur to affect the formation of amyloid-like aggregates, we investigated their effect on insulin fibrillation, a common approach for simulating amyloidosis events. HI was incubated under conditions that promote aggregation in the absence or presence of increasing concentrations (5–100 μg/mL) of curcumin or V-Cur, and the level of amyloids was determined with the specific dye ThT. The results of the fluorometric analysis are given in Figure 3. As it is evident, both substances efficiently inhibited insulin aggregation, but V-Cur complex proved to be a more potent inhibitor. Both curcumin and V-Cur inhibited insulin aggregation in a dose-dependent manner. Curcumin at the lowest employed dose of 5 μM inhibited aggregation by almost 11% and achieved a maximum inhibition of −72% at the concentration of 100 μΜ. On the other hand, the V-Cur complex is a significantly more potent inhibitor, as at a concentration of only 5 μM inhibited aggregation by 71%, while at 40 μM it achieved an approximately 98% inhibition, with a calculated EC_50_ value of 2.03 µM (95% CI: 1.69 to 4.12 μΜ). The respective EC_50_ value for curcumin was 44.06 µM (95% CI: 27.17 to 78.46 μΜ).

### 2.5. Molecular Docking Calculations on Human Insulin (HI)

**Figure 4:**
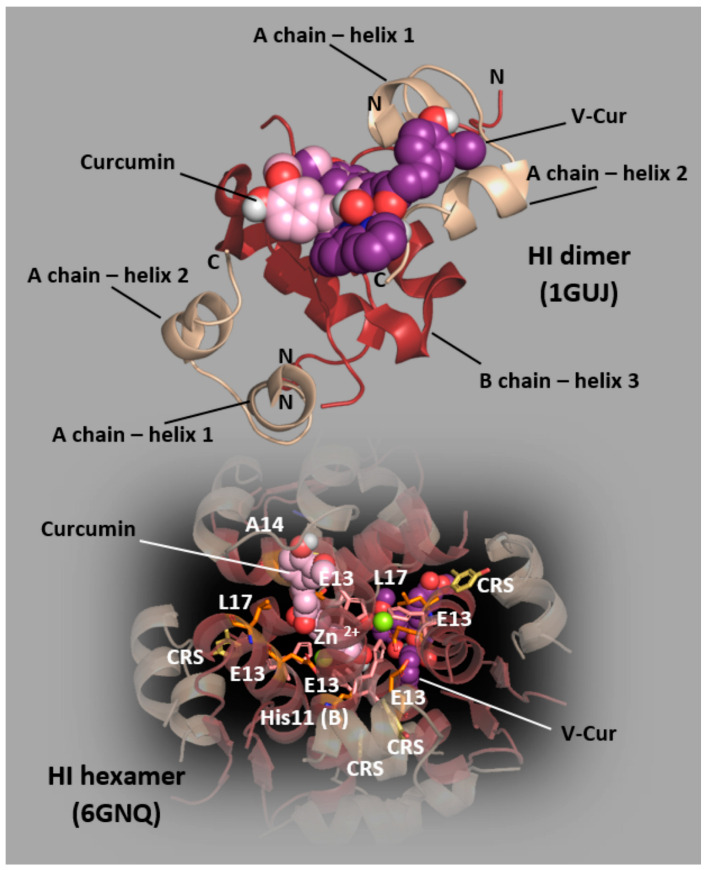
Docking pose orientation of curcumin and V-Cur in the crystal structure of dimer (PDB ID 1GUJ) and hexamer (PDB ID 6GNQ) HΙ target proteins. In the hexameric structure of HI are also illustrated the six chain-stabilizing Zn^2+^ ions, the co-crystallized meta-cresol (CRS, depicted in gold sticks), and some critical to self-assembly and aggregation resides of HI (represented in stick mode colored in orange). Both HI proteins’ structures are depicted as cartoon colored in wheat and firebrick for A and B chains, respectively. Curcumin and V-Cur are rendered in sphere representation colored according to atom type in light pink and violet purple, respectively. The two Zn ions co-crystallized in the hexameric structure are depicted in sphere representation in lemon color and are shown to be connected with polar contact with Nε2 of His10 in the three double chains (A and B). Heteroatom color code: V: grey, N: blue, and O: red. Molecular docking simulations of both ligands were performed individually. Hydrogen atoms are omitted for clarity. The final structure was ray-traced and illustrated with the aid of PyMol Molecular Graphics.

To study the mechanism of the inhibition of HI fibrillation, a molecular docking approach of both curcumin and V-Cur on dimer and hexamer HΙ (1GUJ and 6GNQ PDB IDs, respectively) was employed. From the binding affinity values summarized in Table 1, it is deduced that V-Cur exhibits higher binding affinity for HI compared to curcumin. The binding architecture of best binding poses (best-fitted anchorage) of both curcumin and V-Cur docked in the two HI proteins are depicted in Figure 4.

The best binding pose of both curcumin and V-Cur compounds is located in the interface between chains A and B of the dimer protein interacting with significant residues of HI. This could play a crucial role in the process of insulin fibrillation. From the docking poses of both molecules in the hexameric HI protein, it derives that both curcumin and V-Cur are positioned between the two Zn^2+^ ions in the center of the protein, in a cavity formed between the six symmetry-related chains which stabilize the proteins’ structure. It is obvious that the pocket could accommodate both Zn ions and curcumin and V-Cur at the same place. The amino acid residues contributing to the stabilization of curcumin and V-Cur in the binding cavity of HI protein are Ser9, His10 and Glu13. His10 residues seem to be crucial for the binding of the docked molecule, as previously suggested [35]. Curcumin and V-Cur binding architecture shows that both molecules occupy the same place as the Zn^2+^ ions in the native HI protein (PDB ID: 6GNQ). The hexameric structure of HI protein contains six chains and is stabilized with two Zn ions connected with three symmetry-related histidine molecules (His10) (Figure 4). Binding interaction architecture of curcumin and V-Cur in the ligand-binding site of dimer (PDB ID 1GUJ) and hexamer (PDB ID 6GNQ) HΙ target proteins are depicted in Supplementary Figure S7.

Dimer and hexamer formations lead to the burial of hydrophobic surfaces on the insulin molecule, which might otherwise render insulin vulnerable to random aggregation interactions. The partial unfolding of a monomeric intermediate is believed to be the intermediate stage of the fibrillation process. The unfolding of insulin structure is believed to expose hydrophobic regions of the peptide that eventually trigger its aggregation. The binding of curcumin and V-Cur with residues such as Q15, N18, Y19 of A chain and Y14, L15 of B chain (V-Cur) and N18, Y19 of A chain (curcumin) may play a role in the possible inhibition of HI fibrillation by both molecules, since these residues may be involved in the formation of the spine of insulin fibrils, attenuating thus the fibril formation. Upon fibrillation, the molecule of insulin undergoes structural changes from a predominantly α-helical state to a β-sheet rich conformation. Dimer formation is facilitated by β-strand formation between residues B24–B28 of the two monomers (sequence FFYTP). This FFYTP segment is shown (Figure 4) to be in close proximity to both curcumin and V-Cur, stabilized by several contacts. It is known that the monomerization of HI gives rise to the formation of insulin fibrils. This stabilization of the dimer structure by curcumin and V-Cur could hinder the monomer formation resulting in reduced fibrillation. More details on interaction between curcumin or V-Cur with HI are also provided in the Supplementary Materials.

### 2.6. V-Cur Inhibited the LPS-Induced Increase of iNOS Levels

**Figure 5:**
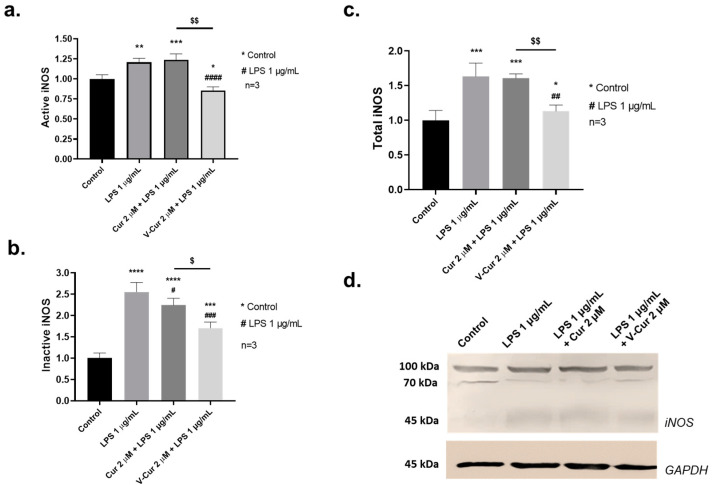
Levels of (**a**) active inducible NO synthase (iNOS) (100 kDa), (**b**) inactive iNOS (50 and 75 kDa), and (**c**) total iNOS levels, after 24 h of treatment with LPS 1 μg/mL, in the presence or absence of 2 μΜ of curcumin or V-Cur complex, in mixed cultures of primary neurons-microglia. iNOS levels were determined by Western blotting (**d**). Glyceraldehyde-3-phosphate dehydrogenase (GAPDH) was employed to verify equal loading. The density of the blots was semi-quantified with ImageJ 1.54. Results are presented as fractional changes in comparison with the control sample and are the mean (±SD) of three independent biological experiments. One-way ANOVA was employed to compare untreated or LPS-treated samples. Statistical significance when compared with: * untreated sample (control); # LPS-treated sample; $ curcumin-treated: * *p* < 0.05; ** *p* < 0.01; *** *p* < 0.001; **** *p* < 0.0001; # *p* < 0.05; ## *p* < 0.01; ### *p* < 0.001; #### *p* < 0.0001; $ *p* < 0.05; $$ *p* < 0.01.

One of the main inflammatory factors induced by LPS is iNOS. Therefore, we determined the effect of LPS (1 μg/mL) on iNOS levels in the co-culture as well as the possible protective effect of either curcumin or V-Cur. Co-culture cell lysates were immunoblotted with the anti-iNOS antibody, and three different bands were quantified (Figure 5a–c), based on the corresponding Western blotting (Figure 5d): a band of approximately 100 kDa, corresponding to the active form of iNOS, and two more bands around 70 and 50 kDa, which correspond to the proteolytically inactivated products of iNOS [36]. The bands of the active and inactive iNOS were quantified and their levels are presented in Figure 5a, and Figure 5b, respectively. Total iNOS levels (determined as the sum of active and inactive iNOS) are presented in Figure 5c.

LPS significantly increased levels of both active (1.21-fold change, *p* = 0.002) and inactivated (2.55-fold change, *p* < 0.0001), and consequently total iNOS (1.63-fold change, *p* = 0.002). Co-treatment of cells with LPS and curcumin moderately inhibited the induction of the inactivated fraction of iNOS (2.24-fold change, *p* = 0.05) but had no effect on the active iNOS levels, when compared to either control or LPS-treated cells. However, V-Cur significantly reduced both active (0.86-fold change, *p* < 0.0001) and inactive (1.7-fold change, *p* = 0.002) iNOS levels compared to LPS-treated cells. These effects are also reflected in total iNOS levels, when comparing samples co-treated with V-Cur and LPS to those treated only with LPS (1.13-fold change, *p* = 0.0016). Noticeably, V-Cur performed as a more effective inhibitor of LPS-mediated induction than pure curcumin, whether assessing the active (*p* = 0.0016), inactive (*p* = 0.0124) or total (*p* = 0.0016) fraction of the enzyme.

### 2.7. Molecular Docking Calculations on iNOS

To further explore the anti-inflammatory role of curcumin and V-Cur, molecular docking simulations with curcumin and V-Cur were conducted against iNOS. The goal was to propose a mechanism for either curcumin or V-Cur in controlling inflammation induction by LPS.

**Figure 6:**
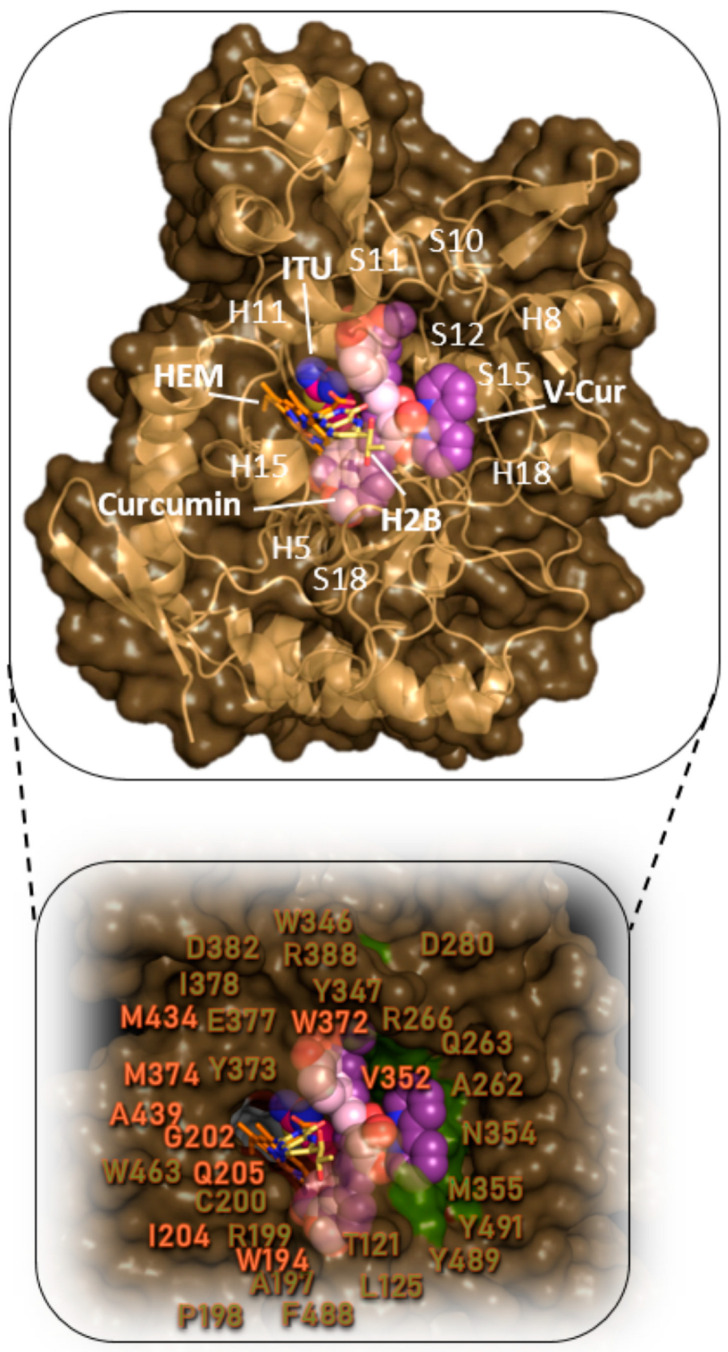
Docking pose orientation of curcumin and V-Cur in the crystal structure of iNOS monomer enzyme (PDB accession number 4NOS). The target protein is illustrated as cartoon colored in the sand along with a semi-transparent surface colored in the dark sand. Curcumin and V-Cur molecules, as well as the co-crystallized iNOS inhibitor ethylisothiourea (ITU) are rendered in sphere mode and colored according to atom type in light pink, violet purple, and hot pink C atoms, respectively. The co-crystallized molecules heme (HEM) (iron protoporphyrin IX) and H2B superimposed with the docked molecules are rendered in stick representation and colored according to atom type in orange and yellow-orange C atoms, respectively. H4B, essential for the dimerization of the protein, is not shown since it is located farther down the binding cavity, near the dimerization interface. The target protein structure model in the lower panel, depicting in a close-up view of the binding cavity of the target enzyme the architecture of the binding interactions, is illustrated as a semi-transparent surface colored in dark sand with an additional depiction of selected contacting amino acid residues of the binding pocket highlighted in the molecular surface in smudge green (for V-Cur) and white (for curcumin). Binding interaction residues are labeled in white (for curcumin) and smudge green (for V-Cur). Heteroatom color code: V: grey, N: blue, and O: red. Molecular docking simulations of both ligands were performed individually. Hydrogen atoms are omitted for clarity. The final structure was ray-traced and illustrated with the aid of PyMol Molecular Graphics.

The docking results (binding affinity) of curcumin and V-Cur on iNOS, are reported in Table 2. Molecular docking simulations showed that curcumin and V-Cur possess good binding affinity towards iNOS, with a binding affinity of −10.7 kcal/mol (Glide score). It is thus deduced that V-Cur exhibits higher binding affinity (better binding capacity) for iNOS compared to curcumin. The best-fitted docking pose of each molecule on the crystal structure of iNOS is depicted in Figure 6. Binding interaction architecture of curcumin and V-Cur in the ligand-binding site of iNOS, TNF-α, and IL-1β target proteins are illustrated in Supplementary Figure S8 and Supplementary Table S2. More details on the interaction of curcumin or V-Cur with iNOS are provided in the Supplementary Materials.

Both curcumin and V-Cur have the potential to bind in a narrow cleft within the catalytic domain of iNOS (Figure 6). Both Cur and V-Cur can be anchored in the active site of iNOS in the same place occupied by heme (HEM) and ethylisothiourea (ITU), an iNOS inhibitor. Curcumin and V-Cur are in a binding pocket enclosed by helices h5, h8, h11, h15, and h18, β-sheets s10, s11, s12, s15, and s18, and loops l120-121, l197-200, l282-283, l262-263, l266, l349-355, l388, and l491-492 (Supplementary Figure S8). Interestingly, interactions of ITU with iNOS (residues Phe369, Asn370, Gly371, Trp372, Glu377), and with the heme molecule, share similarities with part of the curcumin and V-Cur contacts, indicating the capacity of both to act as iNOS inhibitors. V-Cur, due to its bulkier size, is found to contact more residues of the binding site compared to curcumin, while surprisingly, it adopts an orientation such that it enters the binding groove by its whole structure in a 45-degree angle to the heme molecule. At the upper and lower part of Figure 6 is depicted the best binding pose of curcumin and V-Cur superimposed with the co-crystallized inhibitor ITU and the heme molecule, showing that both curcumin and V-Cur can be accommodated in the binding cleft parallel to the heme group. Thus, both compounds facilitate several contacts, being able to contribute to the stabilization of the molecules in the binding pocket.

The binding interactions of curcumin and V-Cur with iNOS residues of the binding cavity are reported in Supplementary Table S2 and illustrated in Supplementary Figure S8. These binding contacts involve hydrogen bond, polar, π-polar, π-anion, π-cation, π-alkyl, and hydrophobic interactions. Further stabilization of the docked molecules inside the catalytic site is achieved with the formation of π-π stacking interactions between the aromatic rings of curcumin and V-Cur and the tyrosine residues Y347, Y373, Y489, and Y491, tryptophane residues W194, W346, W372, and W463, and the phenylalanine residues F369 and F488. Furthermore, π-cation charged electrostatic interactions were observed between the anisole aromatic rings of curcumin and V-Cur and the nitrogen atom of heme, π-anion between carboxyl oxygens of heme with anisole rings of curcumin and V-Cur, and π-alkyl hydrophobic interactions between M355/Cε and the anisole rings of curcumin and V-Cur.

### 2.8. Curcumin and V-Cur Inhibited the Induction of IL-1β and TNF-α by LPS

**Figure 7:**
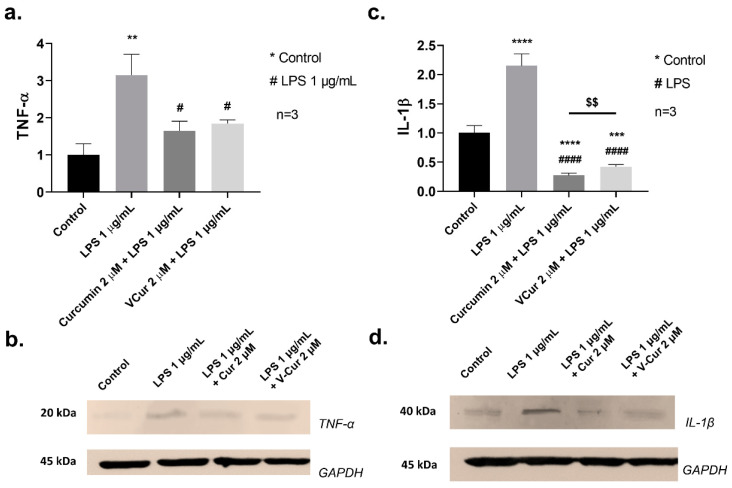
Levels of (**a**) tumor necrosis factor-α (TNF-α), and (**b**) interleukin-1β (IL-1β), in mixed cultures of primary neurons-microglia after treatment with LPS (1 μg/mL) in the presence or absence of 2 μΜ curcumin or V-Cur. Cytokine levels were determined with Western blotting (**c**,**d**). Glyceraldehyde-3-phosphate dehydrogenase (GAPDH) was employed to verify equal loading. The density of the blots was semi-quantified with ImageJ 1.54. Results are presented as fractional changes in comparison with the control sample and are the mean (± SD) of three independent biological experiments. One-way ANOVA was employed to compare untreated or LPS-treated samples. Statistical significance when compared with: * untreated sample (control); # LPS-treated sample; $ curcumin-treated: ** *p* < 0.01; *** *p* < 0.001; **** *p* < 0.0001; # *p* < 0.05; #### *p* < 0.0001; $$ *p* < 0.01.

The cellular levels of TNF-α and IL-1β, two crucial pro-inflammatory mediators of the response to LPS, were also determined (Figure 7a and Figure 7c, respectively) by Western blotting analysis (Figure 7b and Figure 7d, respectively). LPS increased TNF-α (3.15-fold change, *p* = 0.0023) and IL-1β levels (2.15-fold change, *p* < 0.0001) in comparison with untreated samples. Both curcumin or V-Cur inhibited the induction of TNF-α (1.65-fold change, *p* = 0.0155 and 1.85-fold change, *p* = 0.0291) and IL-1β levels (0.27-fold change, *p* < 0.0001 and 0.42-fold change, *p* < 0.0001) by LPS. Moreover, curcumin and V-Cur also reduced the basal levels of the pro-inflammatory marker IL-1β (*p* < 0.0001 and *p* = 0.0004, respectively). Finally, the data indicate that curcumin is a more effective inhibitor of IL-1β induction by LPS than V-Cur (*p* = 0.0092).

## 3. Discussion

Microbes and LPS of Gram-negative bacteria are considered potential co-factors in sporadic AD. For instance, several diseases like periodontitis, Lyme disease, syphilis, leptospirosis, and others, have been associated with increased risk for AD [37,38,39]. Additionally, patients suffering from AD or patients at the prodromal stage of mild cognitive impairment (MCI), present altered gut microbial flora [40,41], while blood levels of LPS have been positively correlated with markers of inflammation, neurodegeneration [42,43] and dysbiosis in these patients [43]. Therefore, LPS are considered potent inducers of neuroinflammation and crucial signaling pathways implicated in the pathophysiology of MCI and AD.

Neuroinflammation is a pathological hallmark of neurodegenerative diseases like AD. However, it is still disputed whether the neuroinflammatory processes occur in favor of remedying or worsening these conditions [44]. Administration of LPS has been widely employed for the experimental induction of neuroinflammation both in vitro, in mixed neuron-glia cultures [45,46,47] and in vivo [48,49]. Indeed, LPS induce neuropathological alterations in laboratory animals that mimic those observed in patients with AD or other neurodegenerative conditions [50,51]. Mixed cultures of neuronal and glial cells simulate the in vivo conditions more effectively, compared to glia-free cultures where ambiguous results have been obtained following exposure to LPS. These data indicate that neurons alone are not able to effectively trigger neuroinflammatory responses [52]. Therefore, in the present study, co-cultures of neurons with glial cells were employed to study the ability of LPS to induce neuroinflammation.

Toxic effects of LPS are mainly attributed to TLR4 signaling in microglia, which ultimately promotes the expression of IL-1β, IL-6, TNF-α, and iNOS [53]. Overproduction of NO by iNOS leads to the extensive formation of neurotoxic peroxynitrite reactive species, which subsequently lead to neuronal damage by inhibition of mitochondrial respiration, caspase-mediated apoptosis, and glutamate-induced excitotoxicity [54]. In addition, NO-mediated nitration of Aβ may be a crucial primal step in plaque formation, as it enhances Aβ aggregation [55]. Consequently, important neuroinflammatory markers including iNOS, TNF-α, and IL-1β were monitored in the current study, along with the crucial AD-related marker APP, after administration of LPS in mixed neuron-microglia cultures.

LPS significantly increased APP levels, with a maximal induction demonstrated at 1 μg/mL of LPS (Figure 1a). Thus, LPS mediates a more potent inductive effect at low concentrations. This could be attributed to the toxic effect of LPS at high concentrations, resulting in decreased cell viability or metabolism, as previously suggested [56]. More studies are required to further investigate such an effect. Increased APP expression is linked to increased Aβ_1-42_ production in patients with inherited AD as well as in AD animal models [57,58]. Therefore, the induction of APP indicates the potency of LPS to induce amyloidosis in our experimental system. This finding is in agreement with previous experiments where APP levels and amyloid aggregation were increased in the hippocampus and cerebral cortex of LPS-injected mice [59,60,61], in LPS-treated astrocytes, microglia, and neuronal cultures [60,61]. Increased Aβ_1-42_ and APP levels have also been identified in the brains of rat mothers and their offspring, as a result of LPS injection on the 17th day of pregnancy [62]. In addition, high expression of APP has been documented in microglia, where it may act as an innate defense factor against HIV-1 that restricts viral release. However, APP processing has been incriminated for Aβ_1-42_ overproduction and neurodegeneration [7]. Although in the co-culture system presented in the present study we cannot safely determine the origin of APP, we can speculate that it is mainly the neuronal cells that contribute to its overproduction. Neuronal cells produce APP, and they constitute a large percentage (90%) in the co-culture system.

As microbes and several of their components (including LPS and DNA) have been detected in senile plaques of AD patients [63,64,65,66], and senile plaques of Aβ are surprisingly like bacterial biofilms [67], another hypothesis has emerged proposing an antimicrobial role of the Aβ peptide, by acting as a “trap-molecule” against infectious agents [6]. Additionally, Aβ peptide presents a microbicidal effect against a variety of clinically significant microorganisms [68]. The results presented here support this hypothesis, implying that *APP* gene transcription may be induced due to a microbial insult (e.g., LPS endotoxins), and thus could be employed in the defense against these agents. Interestingly, increased levels of LPS in cerebrospinal fluid (CSF) and serum or of bacterial rhamnolipids in saliva, significantly correlate with Aβ levels in the CSF and serum of patients suffering from AD or MCI [42,69].

The upregulation of iNOS is documented in the AD brain [70,71] and in the hippocampus and the frontal cortex of mice injected with LPS [61,72], while iNOS knock-out mice exhibit low microglial activation and improved cognitive scores [72]. In the current study, both active and inactive forms of iNOS and subsequently total iNOS levels, were significantly increased by LPS. iNOS and NO titers are increased in vitro because of exposure of microglia or astrocytes to LPS [61], further supporting the central role of LPS on the induction of iNOS activity. Additionally, proteolytical cleavage of iNOS by calpain I is significant for its inactivation [36]. Dysregulated Ca^2+^-homeostasis in AD leads to uncontrolled overactivation of calpains and thus to overproduction of Aβ, p-tau, and neuronal death [73]. Therefore, the increased levels of inactivated iNOS in the current study could be attributed to calpain overactivation by LPS. This is further reinforced by previous in vivo studies, where increased expression and activation of calpain were observed in mice [74] and rats [75] injected with LPS.

TNF-α and IL-1β have a leading role in the induction of neuroinflammation and are considered the main cytokine response to LPS [76,77]. TNF-α is an important pleiotropic factor involved in host defense, inflammation and apoptosis, and has a crucial part in extending the duration of inflammatory processes, while IL-1β is synthesized by microglia and induces inflammatory cascades in response to insult or injury [78,79]. While it is still unclear whether the release of these cytokines is a result of neurodegenerative pathology, or whether these inflammatory agents induce or exacerbate AD-linked hallmarks, it is however verified that both TNF-α and IL-1β induce neuronal damage, synaptotoxicity, and apoptosis [77]. In the present study, LPS significantly increased the levels of TNF-α and IL-1β in rat mixed neuron-microglia cultures. Indeed, LPS treatment of mixed neurons-glia cultures was previously demonstrated to be able to induce the expression and release of TNF-α and IL-1β [80]. LPS also increase the expression of IL-1α, IL-1β, and TNF-α by microglia and induce the hyperphosphorylation of tau protein in co-cultured neurons [81]. In rodents, peripheral administration of LPS leads to an abrupt increase in the expression of TNF-α and IL-1β in the brain. The elevated TNF-α and IL-1β levels may persist for months, in accordance with microglial activation [82].

To summarize, the primary cell co-culture system employed in the current study responds to LPS and adequately reproduces in vitro the neuroinflammatory profile of AD. Therefore, this co-culture system can be used for the identification of compounds with a protective, beneficial effect against AD phenotype. Curcumin, a natural antioxidant, and a V(IV)-curcumin complex (V-Cur) were evaluated as possible therapeutic agents against LPS-induced neurotoxicity in this co-culture system.

Decreased Aβ production, inhibition of Aβ aggregation, and/or promotion of Aβ clearance are the main goals of anti-amyloid therapies against AD [14]. Curcumin dysregulates the maturation processes of APP and inhibits the activity of BACE1 thus preventing Aβ production [83,84,85]. In addition, low μΜ or even nM concentrations of curcumin and its analogs inhibit amyloid aggregation and promote fibril disassembly in vitro [32,86]. Thus, curcumin is a viable candidate for AD management, as it crosses the BBB in vivo and binds to senile plaques in the brain, as demonstrated previously in aged rat model brains, reducing amyloid burden [32,87]. Moreover, the positive effects of curcumin, and especially its modified, bioavailable analogs, on neuroinflammatory and neurodegenerative conditions, have been documented [88]. Clinical trials employing curcumin supplements and nanoformulations have indicated the ability of curcumin to reduce inflammatory markers like IL-6 and C-reactive protein in the circulation and the expression of TNF-α, while they also appease oxidative stress, as demonstrated by reduced blood malondialdehyde and elevated glutathione levels [89]. In addition, in another trial, a daily uptake of 180 mg of bioavailable curcumin improved the cognitive abilities of patients (visual memory, attention, long-term retrieval) and prevented neurodegeneration, as determined by amyloid plaques and tangles [90].

The results presented in the current study indicate the anti-amyloid efficacy of both curcumin and V-Cur, both regarding the expression of APP, as well as the anti-aggregation effect of both compounds against HI self-fibrillation. However, V-Cur, but not curcumin, blocked the increase in APP levels produced by mixed neuron-microglia cultures challenged with LPS (Figure 1c). In addition, curcumin and V-Cur were able to impede amyloidosis, as determined by the insulin fibrilization assay, but V-Cur appears to be more effective than curcumin, as significantly lower doses of V-Cur than curcumin were required to mediate this effect (Figure 3). The inhibitory and disaggregating effect of curcumin on HI fibrils was also documented in a previous study [91]. The anti-amyloidogenic effect of curcumin and V-Cur was further supported by In silico studies, which indicate that both molecules can bind to HI, APP, Aβ_1-42_ monomer, and preformed amyloid fibrils. In silico data parallel the results received from the in vitro study on the co-culture system, as V-Cur imposes a greater effect on the levels of these proteins than curcumin (Table 1). However, further investigation is needed to gain insight into the mechanism of action of these compounds and to establish their role in the inhibition of Aβ formation or aggregation.

In silico studies have brought forth some thoughts regarding the mechanism of anti-amyloid activity of both curcumin and V-Cur. The self-assembly of HI to form amyloid fibrils has been widely studied since it plays a crucial role in regulating amyloid fibrils. It seems that both curcumin and V-Cur inhibit HI-amyloid formation by increasing the resistivity of the amyloidogenic dimer insulin protein to thermal unfolding. It is noteworthy that compounds with a similar structure to that of curcumin, such as rosmarinic acid [92] and its derivatives [93], present common binding contacts (I2, Y19, C20, N21, F25, T27) with curcumin, after docking on the dimer HΙ (PDB ID: 1GUJ).

Regarding Aβ peptide, In silico studies suggest that curcumin or V-Cur interact with the central hydrophobic core recognition sequence KLVFFAED (A*β*_16-23_) of amyloid peptides, as well as the sandwiched β-sheets of the amyloid fibers by forming hydrogen and π bonds (Supplementary Figures S5 and S6). Thus, they could block amyloid aggregation and disrupt fibril structure. Similar interactions of curcumin with Aβ peptide and fibrils have already been suggested [94,95]. Also, binding to the hydrophobic core motif can enhance the disruption of fibril formation, induce fibril disassembly or both because A*β* oligomerization is controlled by the initial interaction of hydrophobic side chains in this central self-interacting hydrophobic core [96,97,98]. In addition, V-Cur and curcumin seem to interact with the crucial site of APP targeted by proteases, and thus possibly restrict its proteolytic processing and further plaque formation, thus offering another insight into the anti-amyloidogenic properties of curcumin and V-Cur.

While curcumin reduced the levels of inactivated iNOS compared to LPS-treated cultures, basal levels could not be reverted. On the other hand, V-Cur was a more potent inhibitor of iNOS expression (Figure 5). Curcumin can block the production of iNOS and NO from rat microglia. Moreover, curcumin inhibits the induction of iNOS in rat brain after LPS administration [99]. Also, curcumin inhibits calpain-mediated proteolysis in rats injected with LPS [100,101], suggesting a possible mechanism for the altered inactivation of iNOS in the present study. The alleviating effects of curcumin on iNOS levels were not reproduced in the present study. However, it should be noted that an exceptionally low dosage (2 μΜ) of curcumin was employed in order to be compared with the strong bioactive V-Cur, which was administered in low concentrations [28,29]. Also, in our experimental system, curcumin was administered to the co-cultures 30 min prior to the addition of LPS. This 30-min pre-treatment period is relatively short compared to the longer pre-treatment periods suggested in other studies [99]. The In silico studies performed here further support that V-Cur can act as an inhibitor of iNOS (Figure 6), hindering NO production, and thus the deleterious effects of LPS-induced inflammation and oxidative stress. Summarizing, the current results underline the superiority of V-Cur in terms of iNOS inhibition, when compared to pure curcumin. The significant reduction in all forms of iNOS (active, inactive and total) mediated consistently by V-Cur indicates the anti-inflammatory role of V-Cur and was further reinforced by the In silico results.

The anti-inflammatory effect of curcumin and V-Cur was further determined by measuring the levels of the crucial inflammatory factors IL-1β and TNF-α in mixed cultures challenged with LPS. Both compounds retained the basal levels of these cytokines, and curcumin had a stronger inhibitory effect than V-Cur on the levels of IL-1β (Figure 7). Curcumin is a strong suppressor of TNF-α and IL-1β expression and secretion from rat glia treated with LPS [102,103,104]. In addition, curcumin improves the viability of microglial cells challenged with a combination of LPS and rotenone—an oxidative stress inducer [103].

Despite the promising results of the present study that suggest an anti-inflammatory and anti-amyloidogenic role of curcumin and V-Cur, the experimental system presented herein is unavoidably with limitations. Undoubtedly, the co-culture system simulates accurately the actual environment of the brain parenchyma. For instance, astroglial cells, which are crucial players in inflammatory reactions, were not included in the current study. In addition, only cerebellar neurons were introduced in the mixed cultures. Moreover, the observed molecular changes should also be complemented with quantitative real-time PCR experiments in the future, to verify and compare expression changes of the analyzed markers with their respective protein levels and thus can help us elucidate the molecular mechanisms implicated and the underlying signaling pathways. Future studies employing primary cells from other CNS regions, or even brain organoids and animal models will surely offer more insight on the observed molecular events, pharmacokinetics and bioavailability of V-Cur in comparison with curcumin.

## 4. Materials and Methods

### 4.1. Materials

Hank’s Balanced Solution (HBSS) #P04-32500, Neuropan basal medium #P04-00900, DMEM with low-glucose (1 g/L) #P04-05551, and fetal bovine serum (FBS) #P30-3031 were purchased from Pan-Biotech (Aidenbach, Germany). Curcumin #8.20354 was purchased from Sigma-Aldrich (St. Luis, Darmstadt, Germany) and used without any further purification, while V-Cur complex was synthesized as previously described [28]. Hank’s Balanced Solution Calcium- and Magnesium-free (HBSS-CMF) #14185052, B-27 supplement #17504044, antioxidant-free B-27 supplement #10889038, GlutaMax #35050038, trypsin in phosphate-buffered saline (PBS) #15050065, Penicillin-Streptomycin (PS) #15140122 and Dulbecco’s PBS #70011044 were acquired from Gibco-Thermo Fischer Scientific (Waltham, MA, USA). Papain #1071440025 and dimethyl sulfoxide (DMSO) #D8418 were from Merck (Darmstadt, Germany). Deoxyribonuclease I (DNase I) #DN25, gentamicin #G1272, amphotericin β #A2942, and LPS #L2630 were purchased from Sigma-Aldrich (St. Luis, Darmstadt, Germany). Poly-L-Lysine (PLL) #33225.01, was acquired from SERVA Electrophoresis GmbH (Heidelberg, Germany). Nitroblue tetrazolium (NBT) #A1442 and 5-bromo-4-chloro-3′-indolylphosphate p-toluidine (BCIP) #A1117 were purchased from PanReac AppliChem (Darmstadt, Germany). The 24-well tissue culture plates (TCP) #38017 were from Costar (Corning Inc., Corning, NY, USA) and 175 cm^2^ flasks #83.3912.502 were purchased from Sarstedt (Nümbrecht, Germany). Only sterile double-distilled water was used when needed. All culture media, buffers and equipment used were sterile and verified as endotoxin- and mycoplasma-free from the supplier and used without any further treatment.

### 4.2. Animals and Housing Conditions

Laboratory animals employed in the current study were newborn rats of the Wistar race (mean weight: 3 day rats: 12 ± 1 g, and 6 day rats: 16 ± 2 g), housed in the Laboratory of Breeding and Development of Animal Standards and Biomedical Research, Faculty of Health Sciences of the Aristotle University of Thessaloniki (License number: EL-54-BIO exp-10). Adult animals were kept in cages of 3 in the animal facility room, with a 12 h light/dark cycle, controlled temperature (22 ± 2 °C), and humidity (50%), with free access to food and water. The litter was provided after controlled crossings of 3-month adults rats (mean weight: male rats: 300 ± 20 g, and female rats: 250 ± 10 g), and was not disturbed until sacrifice. From each birth, the whole litter was sacrificed to receive either cerebellar neurons (at 6 days), or mixed cortical glia (3 days), assuming sex homogeneity within each litter. The use of laboratory animals was licensed by the facility’s Protocol Evaluation Committee and the Directorate of Veterinary Medicine of the Region of Central Macedonia, Greece (License Protocol No. 628751 (2811)) and was performed under the supervision of highly experienced veterinarians. All experiments were carried out in accordance with the Helsinki Declaration and the guiding principles of the European Community Council Directive (89/609/EEC) for the care and use of laboratory animals.

### 4.3. Rat Brain Tissue Collection

Neuronal or glial primary cells were isolated from rat brain tissue as previously described [47]. Animals were sacrificed by decapitation, without anesthesia. Both cerebral cortices were excised from 3-day-old neonates to isolate mixed glial cells, whereas the cerebellum was obtained from 6-day-old neonates, for the isolation of neurons. For each preparation protocol, collected tissues were pooled in cold, sterile HBSS-CMF and processed immediately. All procedures were performed under sterile conditions.

### 4.4. Isolation of Primary Cells from Rat Brain

#### 4.4.1. Isolation and Culture of Primary Mixed Glia from Rat Cerebral Cortex

Mixed glial cells were obtained from rat cerebral cortices and cultured as previously described [47]. Cortices were minced and digested with 0.25% (*w/v*) trypsin in PBS, in the presence of DNase I (20 μg/mL), at 37 °C, for 30 min. After incubation, the cells and undigested tissue were allowed to settle, and the upper liquid fraction was discarded. Cells were washed three times with HBSS-CMF and were re-suspended in the growth medium [low-glucose DMEM (1 g glucose/L), 10% (*v/v*) FBS, 1% (*v/v*) PS]. The cell suspension was filtered through a 100 µm cell strainer (#CLS431752, Corning Inc., Corning, NY, USA) and the flow-through was centrifuged at 200× *g*, at 4 °C for 10 min. The cells were resuspended in growth medium and cultured in 175 cm^2^ PLL-coated flasks at 37 °C, 5% (*v/v*) CO_2_ in a humidified incubator. Growth medium was replaced every 7 days, following a wash with PBS, for 2 weeks. On day 14, microglial cells were collected and co-cultured with neurons, as described below. A representative 14-day-mixed glia culture with mature microglia is given in Supplementary Figure S1.

PLL flasks were prepared as follows: cell-culture flasks were incubated for 2 h at room temperature with a sufficient volume of PLL (40 μg/mL) to cover the entire surface. After removal of PLL solution, the flasks were washed with PBS.

#### 4.4.2. Isolation and Culture of Primary Neurons from Rat Brain

Neurons were collected from rat cerebellum and cultured as previously described [47]. Cerebellums were then digested by papain (1.5 mg/mL) in HBSS-CMF, containing 8 μg/mL DNase I and 5 mM MgCl_2_, at 37 °C, for 30 min (1 mL digestion solution was employed for brain tissue from 2 neonates). After incubation, the mixture was centrifuged for 3 min at 200× *g* at 4 °C, the supernatant was removed, the cells were washed with HBSS-CMF buffer and passed through a 100 µm cell strainer. The recovered cell suspension was centrifuged for 7 min, at 200× *g*, at 4 °C and the cells were resuspended in Neuropan basal medium, supplemented with 2% (*v/v*) B-27, 25 mM KCl, 1.5 mM GlutaMax, 100 μg/mL gentamicin, and 1 μg/mL amphotericin-β.

Approximately 600,000 neurons were plated per well in 24-well cell culture dishes (#38017, Costar) pre-coated with PLL and were incubated at 37 °C, 5% CO_2_ for 1 h. The attached cells were washed twice with HBSS buffer and then fresh culture (growth) medium was added. On day 4, cells were washed with HBSS, and the medium was replaced after substituting the B-27 supplement for antioxidant-free B-27. A representative photograph of an 8-day-neuron culture is provided in Supplementary Figure S1. On day 8, microglia cells were added to establish the co-cultures, as described below.

#### 4.4.3. Isolation of Microglia Cells and Co-Culture with Neurons

Microglial cells were collected from day-14 mixed glia cultures by sharp taps on the side surface of the flasks, followed by gentle agitation of the medium, until most microglial cells were detached, and then cells and media were collected. Microglial cells were harvested by centrifugation for 7 min at 200× *g* at 4 °C and were resuspended in full Neuropan Basal medium, at a final density of 120,000 cells/mL. Approximately 60,000 cells were added as a 0.5 mL suspension in a day-8 culture of neurons grown as described above, to replace half of the medium in the wells of the 24-well plate. Mixed cell cultures were cultured for 2 days, before any following experimentations. A representative photograph of a neuron-microglia mixed culture is provided in Supplementary Figure S1.

### 4.5. Treatment of Mixed Cultures with LPS and/or Curcumin or V-Cur and Preparation of Total Cell Lysates

To induce neuroinflammation, mixed cultures were exposed to 0.1, 1, or 10 μg/mL LPS for 24 h. APP levels were then determined in cell lysates by Western blotting (as described below) and in all subsequent experiments 1 μg/mL LPS was used, based on the highest induction of APP levels found after this treatment. To determine the concentration of V-Cur employed in these studies, we run a set of in vitro antioxidant/anti-inflammatory studies, as well as viability studies in yeast [28] or neuroblastoma-glioblastoma cell cultures (unpublished data). These preliminary experiments indicated that V-Cur at a concentration of 2 μΜ is not toxic and bioactive. The non-toxicity of V-Cur in the concentrations used in the present study was also verified by an MTT assay, as previously described [105] (Supplementary Figure S2). Thus, curcumin or V-Cur (dissolved in DMSO) was added at a final concentration of 2 µM to co-cultures, 30 min. prior to the addition of LPS. The final concentration of DMSO in all samples (including control and LPS-treated cells) was 0.1% (*v/v*). All solutions were prepared fresh before each treatment and were sterilized through a 0.2 μm syringe filter (Minisart NY25 Syringe Filter 17845, Sartorius Stedim Biotech GmbH, Goettingen, Germany). A representative photograph of a neuron-microglia mixed culture treated with LPS and/or curcumin or V-Cur is provided in Supplementary Figure S3. All treatments were replicated in three independent experiments.

Following treatment, cells were washed 3 times with chilled PBS and were lysed with cold RIPA buffer (50 mM Tris-HCl, pH 8.0, 150 mM NaCl, 1% *v/v* Nonidet P-40 (NP-40), 0.5% *w/v* deoxycholate, 0.1% *w/v* SDS), containing 1% (*v/v*) protease inhibitor cocktail (#P8849, Sigma-Aldrich, Darmstadt, Germany), at 4 °C for 30 min. Total cell lysates were recovered, vortexed, centrifuged for 15 min at 13,500 rpm to remove debris, and stored at −20 °C until further analysis. The protein content of the lysates was determined with the bicinchoninic acid assay kit (#71285, Millipore, Darmstadt, Germany).

### 4.6. Western Blotting Analysis

Proteins (30 μg) from each cell lysate were separated by denaturing SDS-polyacrylamide gel electrophoresis (SDS-PAGE), on a 12% (*w/v*) density polyacrylamide gel [106], employing a Labnet ENDURO VE10 device. A solution of 0.3% (*w/v*) Tris-base, 1.44% (*w/v*) glycine, and 0.1% (*w/v*) SDS (pH 8.3) was used as a running buffer. Separated proteins were transferred on a 0.45 µM nitrocellulose membrane, at 4 °C, with a constant current of 200 mA (maximum voltage 100 V) for 45 min, with a transfer buffer (pH 8.3) consisting of 0.3% (*w/v*) Tris-base, 1.44% (*w/v*) glycine, 0.02% (*w/v*) SDS and 20% (*v/v*) methanol.

The membranes were saturated for half an hour with 5% (*w/v*) skimmed milk in PBS, at room temperature, with gentle shaking. For the detection of APP, iNOS, IL-1β, and TNF-α, the following primary antibodies were employed: Anti-APP rat monoclonal antibody (#60342-1-Ig, Proteintech), diluted 1000-times; anti-iNOS rabbit polyclonal antibody (#18985-1-AP, Proteintech), diluted 1000-times; anti-IL-1β rat monoclonal antibody (#sc-52012 Santa Cruz), diluted 500-times; anti-TNF-α rat monoclonal antibody (#sc-52746, Santa Cruz), diluted 500-times. An anti-glyceraldehyde-3-phosphate dehydrogenase (GAPDH) monoclonal rat antibody (#60004-1-Ig, Proteintech) diluted 10,000-times was employed as internal control to confirm equal protein loading and was run once per biological replicate to verify the results of Ponceau S Staining (0.1% *w/v* in 5% *v/v* ethanoic acid) of the transferred proteins on the nitrocellulose membrane (see Supplementary Figure S4). Ponceau staining was applied every time, prior to immunoblotting, to verify efficient transfer and equal protein loading, as previously suggested [107].

All primary antibodies were diluted in PBS, and the incubation was performed for 16 h, at 4 °C. Two alkaline phosphatase (ALP)-conjugated secondary antibodies were used, the anti-mouse IgG (#SA00002-1, Proteintech) (dilution 1:3000) and the anti-rabbit IgG (#SA00002-2, Proteintech) (dilution 1:4000). Membranes were incubated with the secondary antibodies diluted in PBS-T (PBS with 0.05% Tween-20) for 90 min, at room temperature. Each incubation with either primary or secondary antibodies was followed by three washes with PBS-T. The specific protein bands were visualized after immersing the membrane in a buffer containing 100 mM NaCl, 100 mM Tris-HCl pH 9.5, 5 mM MgCl_2,_ and BCIP/NBT (0.5 mM each), and then analyzed with Image J 1.49 (National Institutes of Health—NIH, HΠA) [108].

### 4.7. Insulin Fibrillation Assay

Insulin molecules aggregate into amyloid-like formations which have been used in vitro as a model to assess the anti-amyloidogenic activity of varied factors [109]. The effect of curcumin and V-Cur complex on the formation of amyloid-like aggregates was studied using HI (Actrapid). The formed HI aggregates are detected with the fluorescent dye thioflavin-T (ThT), which presents enhanced fluorescence at 490 nm (excitation at 450 nm) when bound to amyloids [109]. Therefore, HI (200 μM) was incubated in 25 mM HCl (pH 1.6), at 70 °C for 72 h, in the absence or presence of various concentrations of curcumin or V-Cur. The insoluble aggregates that were formed were centrifuged at 13,500 rpm for 15 min, and the fibrils were resuspended in a solution of 20 μM ThT in 50 mM Tris-HCl buffer, pH 7.5. Samples in the absence of ThT were used as blanks. Fluorescence spectra were recorded in a fluorometer (Hitachi F-7000), with excitation at 450 nm and recording of the emission spectrum at 460 to 600 nm, with emission and excitation slit of 2.5 nm. The fluorescence values obtained at 490 nm were plotted to estimate the degree of inhibition of amyloid formation. The experiment was performed in three independent biological replicates, and two technical replicates were also run per experimental cycle.

### 4.8. In Silico Computational Methods (Molecular Docking Calculations)

A series of In silico molecular docking studies were employed to predict the potential interaction of curcumin and V-Cur on HI, APP, Aβ_1-42_ peptide, and Aβ fibrils, target proteins and peptides involved in the neurodegenerative pathway. Details concerning the computation procedures are given in the Supplementary Materials (Section S2).

### 4.9. Statistical Analyses

The statistical analyses and graph construction were performed with GraphPad Prism 8 (GraphPad Software Inc.). All results were deduced from three independent biological experiments. In all provided graphs, bars represent mean value of fractional change in comparison with the control sample ± standard error of the mean (SEM). To examine differences between the untreated and treated groups, standard one-way analysis of variance (ANOVA) was used. To examine differences between treatment with curcumin or V-Cur, a standard unpaired t-test was performed. Half maximal effective concentration (EC_50_) when comparing untreated samples with curcumin or V-Cur, and the corresponding 95% of confidence intervals—CI (95%), were calculated based on the equation: log [x] vs. normalized inhibition (%), with variable slope. Statistically significant results were considered for *p* < 0.05. Notations for statistically significant differences between control (untreated) and treated samples: * *p* < 0.05; ** *p* < 0.01; *** *p* < 0.001; **** *p* < 0.0001.

## 5. Conclusions

The current study demonstrates that rat primary microglial-neuronal co-cultures respond to bacterial LPS insult with the production of inflammatory and amyloidogenic factors, namely APP, iNOS, TNF-α, and IL-1β. Curcumin and V-Cur complex effectively invert this effect. V-Cur was more effective than curcumin regarding the regulation of APP and iNOS levels, while it presented similar inhibitory effects against TNF-α and IL-1β induction. In silico studies offer additional insight on possible molecular interactions between curcumin, V-Cur, and the studied biomolecules. As the possible microbial etiology of sporadic AD is gaining increased attention, hopefully this study will contribute to the restriction of AD’s future prevalence.

## Figures and Tables

**Table 1:** Binding affinity of Curcumin and V-Cur docked on Aβ_1-42_, Aβ fibril, Kunitz protease inhibitor domain (APPI) of APP target peptides, and dimer, hexamer insulin (PDB accession numbers: 1IYT, 2BEG, 1AAP, and 1GUJ, 6GNQ, respectively), with the employment of Glide docking tool with Extra Precision (XP) GlideScore and Molecular Mechanics/Generalized Born Surface Area (MM/GBSA) approach of Schrödinger modeling suite.

Target Peptide (PDB ID)	Binding Affinity (kcal · mol^−1^) Curcumin/V-Cur
A*β*_1-42_ (1IYT)	−8.8/−12.1
A*β* fibril (2BEG)	−12.5/−13.0
APPI (1AAP)	−7.3/−9.4
Insulin dimer (1GUJ)	−6.6/−8.5
Insulin hexamer (6GNQ)	−9.4/−10.1

**Table 2:** Binding affinity of Curcumin and V-Cur docked on iNOS target protein (PDB accession number: 4NOS), with the employment of glide docking tool with extra precision (XP) GlideScore and molecular mechanics/generalized born surface area (MM/GBSA) approach of Schrödinger modeling suite.

Target Protein (PDB ID)	Binding Affinity (Kcal · mol^−1^) Curcumin/V-Cur
iNOS (4NOS)	−8.6/−10.7

## Data Availability

The datasets generated during and/or analyzed during the current study are available from the corresponding author on reasonable request.

## Supplementary Materials

The following supporting information can be downloaded at: https://www.mdpi.com/article/10.3390/ijms26010282/s1. References [33,110,111,112,113,114,115,116,117,118,119,120,121,122,123,124,125,126,127,128,129,130,131,132,133,134,135,136,137] are cited in the Supplementary Materials.
