# Supplementary material for: Evaluation of the Anti-Amyloid and Anti-Inflammatory Properties of a Novel Vanadium(IV)–Curcumin Complex in Lipopolysaccharides-Stimulated Primary Rat Neuron-Microglia Mixed Cultures
Source: Int J Mol Sci. 2024 Dec 31;26(1):282. doi: 10.3390/ijms26010282 (PMC11720140; doi:10.3390/ijms26010282)
Supplement: Supplementary file 1 [file ijms-26-00282-s001.zip › ijms-3350016 Supplementary.pdf]

International Journal of Molecular Sciences
Special Issue: Anti-Inflammatory Strategies and Cell Models for Alzheimer's Disease

Supplementary Materials

Evaluation of the Anti-Amyloid and Anti-Inflammatory Properties of a Novel Vanadium(IV)–Curcumin Complex in Lipopolysaccharides-Stimulated Primary Rat Neuron-Microglia Mixed Cultures

Georgios Katsipis [1,2], Sophia N. Lavrentiadou [2,3,*], George D. Geromichalos [2,4], Maria P. Tsantarliotou [3], Eleftherios Halevas [1,5], George Litsardakis [6] and Anastasia A. Pantazaki [1,2,*]

[1] Laboratory of Biochemistry, Department of Chemistry, Aristotle University of Thessaloniki, 54124 Thessaloniki, Greece; gkatsipis@chem.auth.gr (G.K.); lefterishalevas@gmail.com (E.H.)

[2] Center for Interdisciplinary Research and Innovation, Laboratory of Neurodegenerative Diseases (LND), Thermi, 57001 Thessaloniki, Greece; gerom@chem.auth.gr

[3] Laboratory of Animal Physiology, School of Veterinary Medicine, Aristotle University of Thessaloniki, 54124 Thessaloniki, Greece; mtsant@vet.auth.gr

[4] Laboratory of Inorganic Chemistry, Department of Chemistry, Aristotle University of Thessaloniki, 54124 Thessaloniki, Greece

[5] Institute of Biosciences & Applications, National Centre for Scientific Research "Demokritos", 15310 Athens, Greece

[6] Laboratory of Materials for Electrotechnics, School of Electrical and Computer Engineering, Aristotle University of Thessaloniki, 54124 Thessaloniki, Greece; lits@eng.auth.gr

* Correspondence: slavrent@vet.auth.gr (S.N.L.); natasa@chem.auth.gr (A.A.P.); Tel.: +30-2310-999872 (S.N.L.); +30-2310-997838 (A.A.P.); Fax: +30-2310-997689 (A.A.P.)

Index

Content	Page
**Section S1. Culturing of primary rat mixed neuron-glia cultures**	3
**Figure S1.** Representative photos from rat primary neuronal cells isolated from neonatal brains and cultured in the current study. From left to right:14-day primary microglia, 8-day neurons, and 10-day neurons after mixing with microglia on day 8. Photos were captured from an inverted microscope (200-times final magnification).	3
**S1.1.** Microscopic evaluation of mixed neurons-microglia cultures treated with LPS and/or curcumin or V-Cur	3
**Figure S2.** Viability of mixed cultures in the presence or absence of 2 μM of either curcumin or V-Cur complex, as determined by MTT assay.	4
**Figure S3.** Light inverted microscopy pictures of mixed cultures of primary neurons-microglia isolated from neonatal brains, at 100 X magnification, after being treated with 0.1% (v/v) DMSO (Control sample), LPS 1 μg/ml, co-treated with LPS and curcumin 2 μM, and co-treated with LPS and V-Cur complex 2 μM.	5
**Figure S4.** Ponceau S staining of blotted proteins on nitrocellulose membrane, after electrophoretic separation and subsequent transfer of 30 μg total protein sample from mixed cell lysates.	6
**Section S2. Molecular docking calculations**	7
**S2.1.** ***In silico*** **computational methods**	7
**S2.2. Further information on docking studies**	10
**Table S1.** Binding contact residues of curcumin and V-Cur molecules in the binding site of APPI.	14
**Table S2.** Binding contact residues of curcumin and V-Cur molecules in the binding cavity of iNOS.	15
**Figure S5.** Docking pose orientation of curcumin and V-Cur in the crystal structure of Aβ1-42 monomer peptide (Aβ, containing 1-42 amino acids).	16
**Figure S6.** Docking pose orientation of curcumin and V-Cur in the crystal structure of Aβ17-42 fibril.	18
**Figure S7.** Binding interaction architecture of curcumin and V-Cur in the ligand-binding site of dimer and hexamer HI target protein.	20
**Figure S8.** Binding interaction architecture of curcumin and V-Cur in the ligand-binding site of iNOS.	21

Section S1. Culturing of primary rat mixed neuron-glia cultures

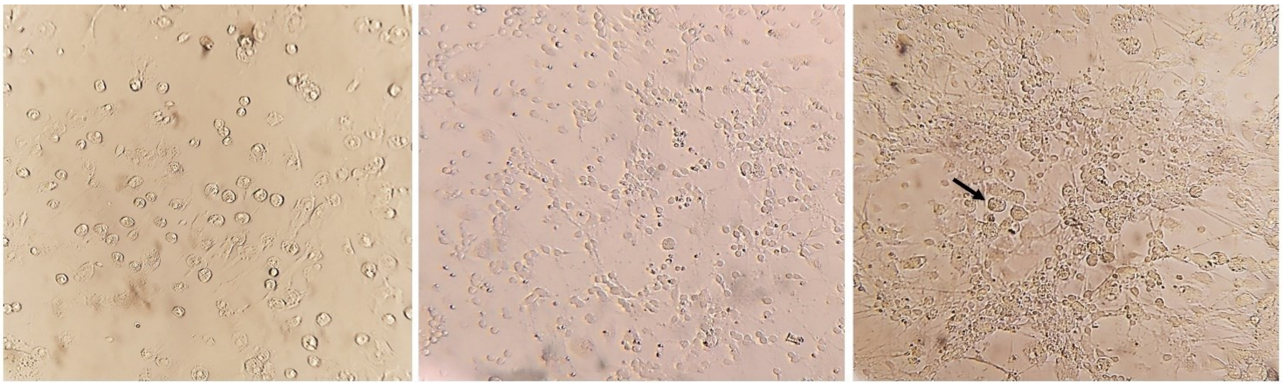

Primary Mixed Glia
Day 14

Primary Rat Neurons
Day 8

Mixed Neuron-Microglia
Day 10

200X

Figure S1. Representative photos from rat primary neuronal cells isolated from neonatal brains and cultured in the current study. From left to right:14-day primary microglia, 8-day neurons, and 10-day neurons after mixing with microglia on day 8. Photos were captured from an inverted microscope (200-times final magnification).

S1.1. Microscopic evaluation of mixed neurons-microglia cultures treated with LPS and/or curcumin or V-Cur

Mixed cultures of rat primary neurons and microglia 24 hours after treatment with LPS in the absence or presence of curcumin or V-Cur were observed under an inverted microscope, at 100x magnification (Figure S2). Exposure of co-cultures to LPS, curcumin, or V-Cur did not elicit any observable alterations in cell morphology. However, LPS significantly reduced the observed neurons that were cultured in the absence of microglia. In contrast microglia were present in higher density in the LPS-treated group compared to the control group (Figure S2). The presence of curcumin or V-Cur inhibited this increase in microglia density. Interestingly, V-Cur was precipitated on extracellular, aggregative, orange formations on the culture plates when observed under an inverted light microscope (Figure S2). Such an effect was also found in some cases for curcumin too. Still, the formations were more dispersed, with weaker coloration.

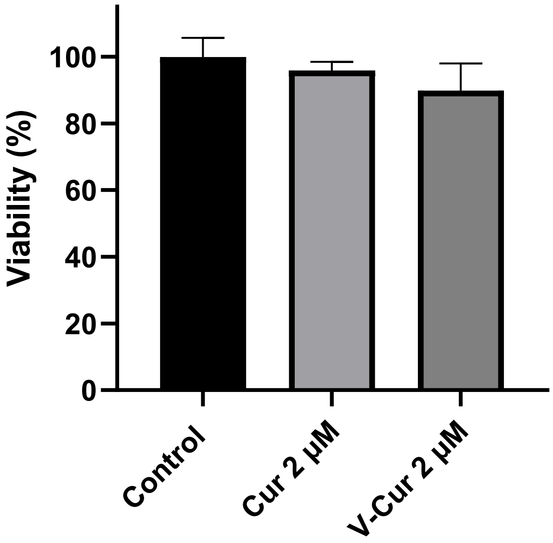

Figure S2. Viability of mixed cultures in the presence or absence of 2 μM of either curcumin or V-Cur complex, as determined by MTT assay. Statistical analysis with one-way ordinary ANOVA. No statistically significant differences have been found.

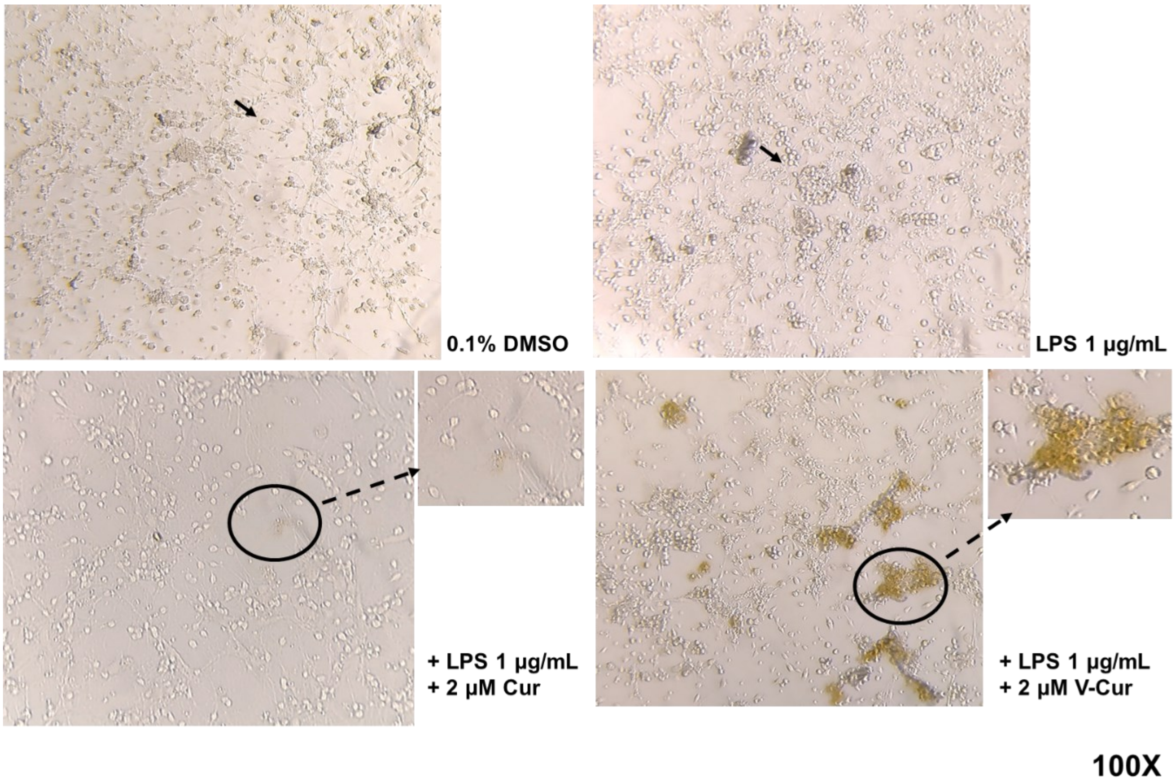

Figure S3. Light inverted microscopy pictures of mixed cultures of primary neurons-microglia isolated from neonatal brains, at 100 X magnification, after being treated with 0.1% (v/v) DMSO (Control sample), LPS 1 μg/ml, co-treated with LPS and curcumin 2 μM, and co-treated with LPS and V-Cur complex 2 μM. Picture insets: enlarged areas from the cultures treated with either curcumin or V-Cur complex, where orange aggregates/precipitates were visualized after 24 hours of treatment.

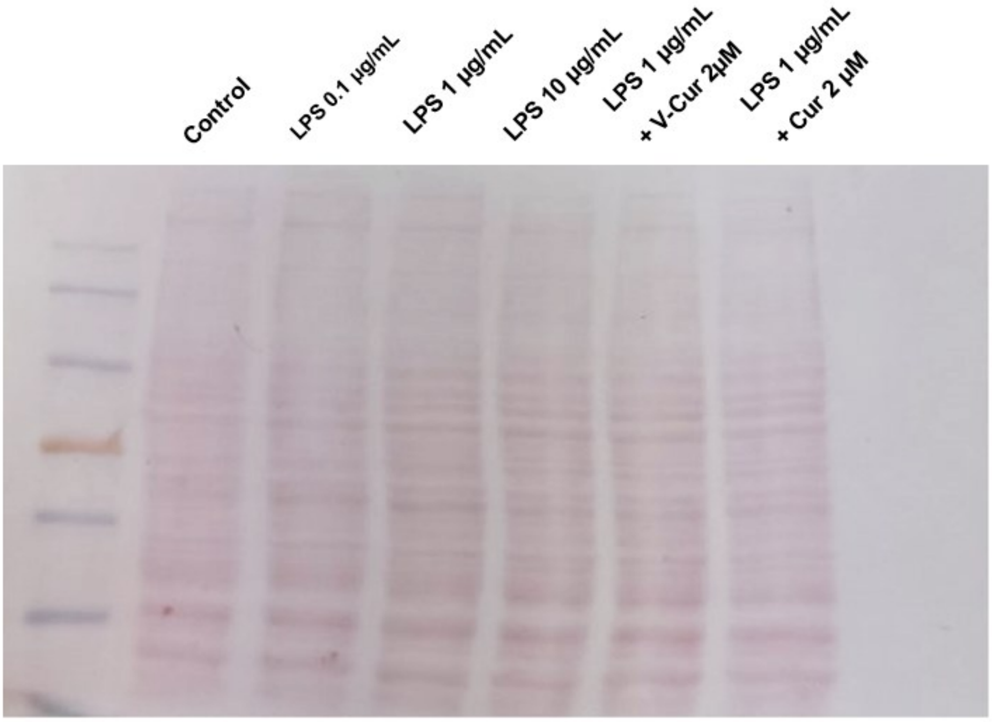

Figure S4. Ponceau S staining of blotted proteins on nitrocellulose membrane, after electrophoretic separation and subsequent transfer of 30 μg total protein sample from mixed cell lysates.

Section S2. Molecular docking calculations

S2.1. *In silico* computational methods

A series of *in silico* molecular docking studies were employed to predict the potential inhibitory activity of curcumin and V-Cur on APP, A$\beta_{1\text{-}42}$, Aβ fibril, dimer and hexamer human insulin and iNOS target proteins and peptides involved in the neurodegenerative pathway. The *in silico* predictive tools that have been employed to study the interactions of the compounds with the selected macromolecules, are Schrödinger, Mercury, ChemBio3D Ultra, Spartan' 14, and PyMol molecular modeling software. The 3-D structure of the synthesized vanadium/curcumin complex was generated from its X-ray crystal structure as a CIF file. Mercury software (http://www.ccdc.cam.ac.uk/) was then used to convert the CIF file to a PDB format file. The structure of curcumin was retrieved by PubChem chemical information resource library at the U.S. National Center for Biotechnology Information (NCBI) (https://pubchem.ncbi.nlm.nih.gov) (CID_969516). 3D conformers of each molecular structure were optimized through energy minimization (MM2 force field method) with the aid of ChemBio3D Ultra v. 14.0.0.117 software suite (CambridgeSoft Corporation) and subsequently PDB files of each structure were generated. The best, most stable (lowest energy) conformation of the molecular model of each compound was detected by geometrical optimization in the gas phase, as implemented in the Spartan '14 Molecular Modeling program suite (Spartan '14 v.1.1.4, Wavefunction Inc., Irvine, CA, USA; www.wavefun.com.). The structures were initially optimized (*via* energy minimization) by conformational search using the Monte Carlo method with the MMFF94 molecular mechanics model, included in the Spartan'14 program suite. Geometry optimization (leading to the most stable conformer with the lowest energy) was accomplished *via* quantum-chemical calculations by utilizing Density Functional Theory (DFT) computations at the B3LYP level of theory with 6-31G*(d,p) basis set to describe the accurate structural and electronic properties of the compounds, implemented by Spartan' 14 program suite.

Molecular docking calculations were carried out on the crystal structures of the target proteins iNOS [Protein Data Bank (PDB) accession numbers: 4NOS] refined at

2.25 Å, respectively. The X-ray crystallographic structures were obtained from the Brookhaven Protein Data Bank (operated by the Research Collaboratory for Structural Bioinformatics, RCSB) [110–112]. iNOS with 427 amino acid length is co-crystallized with iron protoporphyrin IX (heme), 5,6,7,8-tetrahydrobiopterin (H_4B), dihydrobiopterin (H_2B) 2-amino-6-(1,2-dihydroxy-propyl)-7,8-dihydro-6H-pteridin-4-one, ethylisothiourea (ITU), and Zinc ion (Zn^{+2}) [113]. For the docking calculations on iNOS it only the A chain of the protein was used, since chains B, C, and D, are replicates, with co-crystallized ligands bound at the same ligand binding site among the chains. For this reason, from corresponding PDB files it was deleted the data for chains B-D and also the data of the drug referring to these chains.

Molecular docking calculations were also carried out on the solution-state NMR structures of $A\beta_{1-42}$ [114], and Aβ fibril [115] target peptides (PDB accession numbers: 1IYT, 1QWP, and 2BEG, respectively), the crystal structure of the Kunitz protease inhibitor domain (APPI) of APP [33] determined and refined to 1.5 Å resolution (PDB accession number 1AAP), as well as on the dimer crystal structure of human insulin determined to 1.62 Å resolution [116] and the hexamer crystal structure of HI at 2.20 Å resolution (PDB accession number: 6GNQ) [117]

The Schrödinger software suite contains a broad array of computational chemistry tools. In the procedure for molecular docking with the employment of Schrödinger suite both compounds were converted into three-dimensional MOL2 files using Schrödinger Release 2020-3 Maestro Version 11.1 and minimized using LigPrep 3.5 [118] (which can generate several structures from each input structure with various ionization states, tautomers, stereochemical characteristics, and ring conformations to eliminate molecules based on various criteria such as molecular weight or specified numbers and types of functional groups with correct chiralities for each successfully processed input structure), and the OPLS3 (Optimized Potential for Liquid Simulations) [110] force field for the optimization, producing the low-energy isomers of the ligands (Schrödinger, http://www.schrodinger.com). Energy-minimized 3D molecular structures were generated with the employment of LigPrep run from the Maestro utility of the Schrödinger suite. The ligand preparation included 2D–3D conversions, generating variations, correction, verification, and optimization of the

structures. A preparation of receptor and ligand structures was integrated before the actual docking procedure [119]. The crystal structures of the proteins were prepared using the Protein Preparation Wizard [120], in Schrödinger Suite 2020-3 (Schrödinger, LLC, New York, NY) [121,122]. Protein was prepared by adding the hydrogen atoms, optimizing hydrogen bonds, removing atomic clashes, adding formal charges to the hetero groups, and then optimizing at neutral pH. Missing loops and side chains were prepared using Prime version 3.2 [123,124]. Finally, the structure was minimized using the OPLS3 force field. The active site of studied proteins was obtained using the SiteMap tool (version 3.6, Schrödinger) [125], which provides a fast and effective means of identifying potential binding pockets of proteins. SiteMap identifies the character of binding sites using novel search and assesses each site by calculating various properties like size, volume, amino acid exposure, enclosure, contact, hydrophobicity, hydrophilicity, and donor/acceptor ratio. The receptor grid was generated around the active site for effective binding using Receptor grid generation in the Glide (version 5.9) application of Maestro. Once the receptor grid is generated, the ligands are docked to the proteins using the Glide docking tool of Schrödinger (Grid-based Ligand Docking with Energetics) (version 6.8) [126]. Compounds were docked in the binding site of the proteins using the Induced-Fit Docking (IFD) protocol 2020-3 [127–129]. The ligand interactions are shown in the Ligand interaction tool of Maestro (Schrödinger). Waters were deleted with Maestro, the graphical user interface (GUI) of Schrödinger software, before docking. Molecular docking studies were carried out for the best-fitted compounds to the model, while the final selection criteria were compound docking scores and the presence of crucial interactions for binding to the studied proteins [130]. The resulting poses were examined manually, and the most promising ones were redocked with IFD calculations. Poses that pass the initial screens enter the final stage of the algorithm, which involves the evaluation and minimization of a grid approximation to the OPLS-AA nonbonded ligand-receptor interaction energy. Final scoring is then carried out on energy-minimized poses. By default, Schrödinger's proprietary Glide Score [131] multi-ligand scoring function is used to score the poses. The rescoring was performed to calculate and improve binding energy calculations with Prime's Molecular Mechanics-Generalized Born Surface Area (MM-

GBSA) protocol using the VSGB solvation model [132,133]. All complexes showed good docking scores reflecting drug-binding affinities with the studied proteins. PyMol Molecular Graphics System (Schrödinger, LLC. version 2.3.5, www.pymol.org) [134] was used to visualize the molecules and analyze the results of the docking and to construct the molecular models.

S2.2. Further information on docking studies

A$\beta_{1\text{-}42}$

The binding poses of both curcumin and V-Cur (Figure S5) suggest anchorage in the binding pocket with the formation of contacts with the KLVFFAED sequence of the peptide (K16, L17, and F20 for curcumin and K16, F19, F20, and D23 for V-Cur) as well as binding sites before (N-terminal) and after (hinge region) KLVFFAED (V12, V24, I31, and L34 for curcumin, and V24, N27, I31, and L34 for V-Cur). Common binding residues among the two molecules were revealed to be K16, F20, V24, I31, and L34. Similar binding contacts of curcumin and curcumin derivatives have previously reported [135]. Free axial vanadate oxygen of V-Cur is also a hydrogen bond connected to D23 carboxylic Oδ1 (2.1 Å). Further stabilization of V-Cur inside the binding site of A$\beta_{1\text{-}42}$ monomer peptide is achieved by the formation of π-π stacking T-shaped contacts formed between the aromatic rings of F20 and F19 and aromatic C atoms of anisole moiety of curcumin and the 2,2′-bipyridine (bipy) of V-Cur, respectively. Other special interactions of V-Cur involve π-charge electrostatic contacts including π-anion between the negative charge of the pyridine aromatic ring of bipy moiety and the negative charge of carboxylic Oδ2 of D23 (3.0 Å) and π-cation between the negative charge of one of the anisole rings and the positive charge of Nζ of K16 (3.1 Å). Additionally, polar contacts were established between the axial vanadate O and either I31/Cδ1 or V24/Cγ2 (2.9 or 3.4 Å, respectively). Curcumin was found to be anchored in its binding site *via* the formation of π-polar contacts with F20, π-cation with K16/Nζ, π-alkyl with K16/Cε, V12/Cγ2, L34/Cδ2, and V24/Cγ2, and polar contact between the phenolic OH of anisole and I31/Cγ2 (2.4 Å). Furthermore, it has been reported [136] that there are three structural properties of interaction that can destabilize the

assembly of A*β*: 1) Electrostatic interaction between residues D23-K28 forms a hydrophilic region; 2) E22 helps in maintaining the stability between chains of fibrils and helps to remain intact, and 3) Residue in between L17-A21 and A30-A42 from a hydrophobic region which can be the target region to mask and block oligomerization. Most of these binding regions are shown to be common with those of curcumin and V-Cur.

Aβ fibril

The ligand-binding site of molecules depicting the extent of the pocket as determined by the computation process, labeling the critical residues interacting with the molecules, are shown in the upper part of Figure S6. Ligand interactions of curcumin and V-Cur stabilized at the β clusters formed at the concave edge of the Aβ fibril. The docking procedure predicts the formation of a variety of interactions with residues L17, F19, V40, I41, and A42 of all five chains A-E. Stabilization of both anchored compounds may be attributed to H-bond, hydrophobic, and polar contacts. Other special interactions involve π-polar and π-alkyl type hydrophobic contacts with the residues of the binding site. Interestingly, one of the binding contacts of both curcumin and V-Cur, F19, is reported to be one of the key inter-sheet contacts formed between residues of the antiparallel sheets (F19/G38).

Human insulin (HI)

The stabilization of both anchored compounds on the hexamer HI may be attributed to H-bond, hydrophobic, and polar contacts. Other special interactions involve π-polar and π-alkyl type hydrophobic contacts with the residues of the binding site. Binding of curcumin and V-Cur to B chain residues E13, A14, and L17 of the hexamer HI, prevent exposure of hydrophobic residues inhibiting self-assembly and aggregation that leads to fibrillation (Figure S7). Curcumin interacts with E13 of B2 and B3 chains, E13 and A14 of B4 chain, E13 and L17 of B5 chain and E13 of B6 chain. Furthermore, V-Cur found to be connected with L17 of B6 chain (A1-A6 and B1-B6 represent the chains of the hexamer HI). Other binding contacts of curcumin on hexamer HI involve V3, C6, C7, S9, I10, C11, S12, L13, Y14, L16, and E17 of A chain,

and F1, V2, Q4, H5, L6, G8, S9, H10, L11, E13, Y16, L17, V18, C19, G20, E21, Y26, P28, and K29 of B chain. On the other hand, V-Cur interacts with hexamer HI via V3, H5, C7, T8, I10, C11, S12, L13, and Y14 residues of A chain, and F1, Q4, G8, Y16, L17, V18, C19, G20, E21, Y26, P28, and K29 residues of B chain. It seems that several hydrophobic contacts additionally stabilized the anchored curcumin and V-Cur molecules in the binding cavity of the HI protein.

Both curcumin and V-Cur are shown to be anchored in a binding pocket of the dimer HI more closely to one monomer, enclosed by helices 1 and 2 of the A chain, flanking at the other side of the cleft the β-sheet region of B chain. One of the anisole moieties of V-Cur is likely in close proximity to the N terminus of helix 1 of the A chain, while the bipy moiety is near to the C terminus of helix 2. Curcumin interactions with residue of the B chain of the dimer HI include T27/O, N, Oγ1 (2.4, 3.0, 3.7 Å, H-bond), F25 (3.6 Å, π-π stacking sandwich type), and Y26 (3.2 Å) (aromatic ring with the methoxy-O atom of the anisole moiety of the compound). Further stabilization of curcumin in the binding pocket of the dimer HI is achieved with the contribution of π-π T-shaped interactions between the anisole aromatic ring of curcumin and the aromatic ring of Y19 (helix 2 of A chain), π-alkyl type hydrophobic contact between the anisole aromatic ring and I2/Cγ2, Cδ1 (3.2, 3.9 Å) of helix 1 of A chain. Additional stabilization is achieved via polar and H-bond binding of curcumin to N18/O (3.6 Å), Y19/O (3.6 Å), C20 (4.0 Å) of helix 2 of A chain. Further stabilization of curcumin is attributed to π-π displaced interaction of the molecule to F25 of the B chain. Common binding contacts between curcumin and V-Cur include Y26 and T27 of the B chain, I2, N18, Y19, C20, and N21 of A chain (e.g. π-polar contact of bipy moiety of V-Cur with N21/Nδ2 (3.5 Å) and H-bond between one axial vanadate oxygen atom of V-Cur and C20/O (2.7 Å) of helix 2). Other binding contacts of V-Cur include I2, Y14, L15, C20, F25 (π-π T-shaped and displaced interactions), Y26, T27, and P28 of the B chain, and 12, Q5, Q15, N18, Y19, C20, and N21 of A chain. Previous studies revealed a role for some segments of A chain (residues A13–A19) and B chain (residues B9–B19) in conditions favorable for fibril formation [137]

APPI

In Figure 2 of the manuscript is depicted the best-fitted anchorage of curcumin and V-Cur in the active site of APPI, labeling also the critical amino acid residue contacts. The binding interactions are reported in Table S1. The binding contacts of both compounds in the interface between chains A and B of the protein were revealed to be residues Q8, A9, Y22, D24, T26, E27, and F33 of chain A and Q8, T11, D24, V25, T26, P32, F33, and F34 of chain B (curcumin), and Q8, A9, Y22, D24, V25, and T26 of chain A and Q8, A9, T11, Y22, D24, T26, E27, P32, F33, and F34 of chain B (V-Cur). Most of the residues are shown to be common between the two molecules.

iNOS

Binding residues of either curcumin only W194, F369, and W372, or only V-Cur, Y489, and both curcumin and V-Cur M355, found to be common with that of heme (Figure 6 of the manuscript). Furthermore, a binding contact of both docked molecules, E377 located adjacent to the active site, is also forming an essential bidentate interaction by its glutamate group with the inhibitor ITU. The ethyl group of ITU is shown to be packed near the heme and F369 side chain [4], which is a binding contact of curcumin. T463 common binding residue of the monomer near the dimerization interface is located on one side of the pocket (T461 and F476 being the opposite face residues from the other monomer, not shown in the structure).

Further stabilization of both molecules in the binding pocket is achieved with the formation of π-cation and π-anion contacts between the aromatic rings of one of the anisole moieties of curcumin and V-Cur and R388/Nη1 and D382/Oδ1, E377/Oε1 atoms, respectively (Figure S8). Pi-cation interaction is also observed between R266/Nη1 and bipy moiety ring of V-Cur and π-polar between T121/Oγ1 and N354/Nδ2 and bipy ring, and N354/Nδ2 and N263/Nε2 and one of the anisole rings of V-Cur and π-alkyl between A262/Cβ and bipy. Additionally, the N454/NH is found to be involved in a hydrogen bond (H-bond) with one of the pyridine nitrogens of the bipy moiety and one axial vanadate oxygen atom of V-Cur. H-bond contacts were also observed between both hydroxyl anisole oxygens of V-Cur and curcumin and Y373/OH, E377/O, and C200/N, and between anisole O of curcumin and R199/N. Furthermore, both

phenolic and methoxy-O atoms of anisole are hydrogen bonds connected to E377/O (2.1 Å), D382/HO2, Oδ2 (3.2, 3.1 Å), I378/N (3.7 Å), and R388/Nη1, Nη2 (3.5, 2.9 Å). Both molecules are further stabilized in the binding pocket of iNOS with the contribution of π-π stacking, displaced and T-shaped interactions between the anisole aromatic ring and the aromatic rings of W463, F369, Y373, and W346, and the bipy ring and Y491. The free axial vanadate hydroxyl group is also a hydrogen bond connected to Oδ1 of N354 (3.6 Å). Several hydrophobic contacts additionally stabilized the anchored curcumin and V-Cur molecules in the binding cavity of the enzyme.

Table S1. Binding contact residues of curcumin and V-Cur molecules in the binding site of APPI.

**Target protein**	**Binding contact residues (Å)**			
	**Curcumin**		**V-Cur**	
**APPI**	GLN 8A*	2.878	GLN 8A*	4.093
	ALA 9A*	3.414	ALA 9A*	3.646
	TYR 22A*	2.757	TYR 22A*	3.197
	ASP 24A*	2.834	ASP 24A*	3.397
	THR 26A*	3.116	VAL 25A	4.163
	GLU 27A	3.965	THR 26A*	2.850
	PHE 33A	4.036	GLN 8B*	2.963
	GLN 8B*	3.830	ALA 9B	3.323
	THR 11B*	3.076	THR 11B*	3.753
	ASP 24B*	4.013	TYR 22B	1.823
	VAL 25B	3.556	ASP 24B*	2.200
	THR 26B*	2.312	THR 26B*	3.353
	PRO 32B*	3.830	GLU 27B	3.647
	PHE 33B*	3.326	PRO 32B*	2.980
	PHE 34B*	2.895	PHE 33B*	3.302
			PHE 34B*	2.759

*Binding contacts common between curcumin and V-Cur

Table S2. Binding contact residues of curcumin and V-Cur molecules in the binding cavity of iNOS (chain A of iNOS indicate the monomer structure).

Target protein	Binding contact residues (Å)			
	Curcumin		V-Cur	
**iNOS**	TRP 194A	3.579	THR 121A	3.886
	ARG 199A	3.485	ALA 197A	3.320
	CYS 200A	3.964	PRO 198A	3.433
	GLY 202A	3.432	ARG 199A	3.816
	GLN 205A	3.238	CYS 200A	3.550
	SER 242A	4.014	ALA 262A	3.462
	VAL 352A*	3.979	GLN 263A	3.412
	MET 355A*	3.310	ARG 266A	3.124
	PHE 369A	3.303	TRP 346A	2.987
	ASN 370A	2.916	TYR 347A	3.253
	GLY 371A	3.285	VAL 352A*	4.056
	TRP 372A	2.890	ASN 354A	3.586
	TYR 373A*	3.984	MET 355A*	3.452
	MET 374A	3.756	TYR 373A*	2.394
	MET 434A	3.964	GLU 377A*	3.016
	GLU 377A*	4.016	ILE 378A	3.971
	ALA 439A	4.090	ASP 382A	2.278
	TRP 463A*	3.982	ARG 388A	2.945
	TYR 491A	3.758	TRP 463A*	3.910
			PHE 488A	3.551
			TYR 489A	3.777
			TYR 491A*	2.649

*Binding contacts common between curcumin and V-Cur

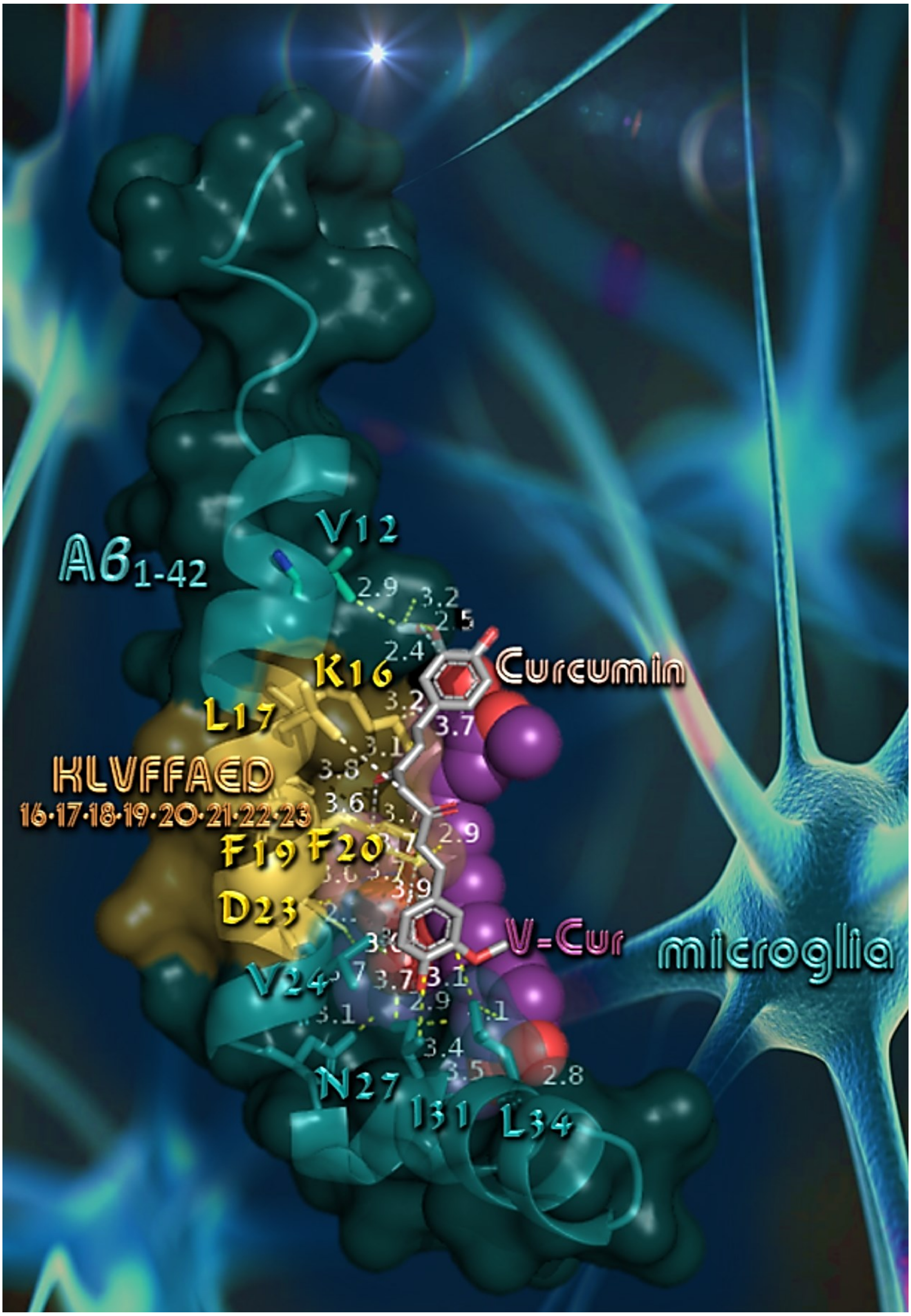

Figure S5. Docking pose orientation of curcumin and V-Cur in the crystal structure of Aβ_{1-42} monomerpeptide(Aβ,containing1-42 amino acids) (PDB accession number 1IYT). Target peptide is illustrated as cartoon colored in deep teal along with semi-transparent surface of the same color. In yellow orange is colored the Aβ central

hydrophobic core Recognition Sequence KLVFFAED (Aβ16-23). Curcumin and V-Cur molecules are rendered in stick and sphere mode, respectively and colored according to atom type in silver, and violet purple C atoms, respectively. The ligand binding site of both molecules in Aβ1-42 monomer peptide depicting the architecture of the binding interactions are also illustrated with additional depiction of selected contacting amino acid residues of the binding pocket rendered in deep teal and yellow orange sticks. Binding interaction residues are illustrated in white (for curcumin) and yellow (for V-Cur). Heteroatom color-code: V: grey, N: blue, and O: red. Molecular docking simulations of both ligands were performed individually. Hydrogen atoms are omitted for clarity. The final structure was ray-traced and illustrated with the aid of PyMol Molecular Graphics.

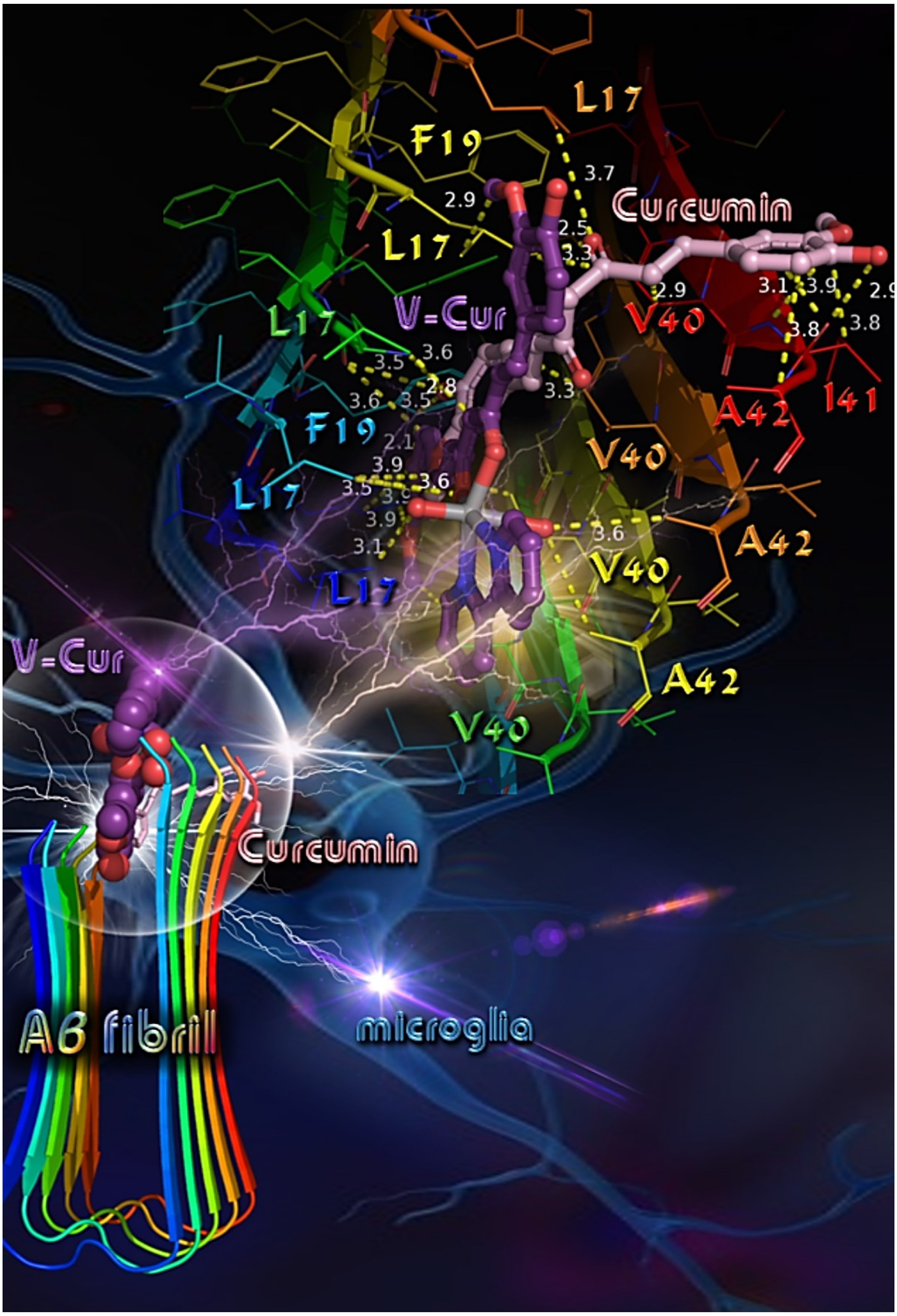

Figure S6. Docking pose orientation of curcumin and V-Cur in the crystal structure of Aβ17-42 fibril and (PDB accession number 2BEG). The model used consists of residues 17-42 (L17-A42) forming a beta-strand-turn-beta-strand motif that contains two intermolecular, parallel, in-register beta-sheets that are formed by residues 17-26 (L17-

S26) (beta1) and 31-42 (I31-A42) (beta2) connected with a loop in a pentamer (chains A-E) construction. Target peptide is illustrated as cartoon colored in rainbow while curcumin and V-Cur molecules are rendered in ball-and-stick mode and colored according to atom type in light pink and violet purple C atoms, respectively. The ligand binding site of both molecules in Aβ17-42 peptide depicting the architecture of the binding interactions is also illustrated with additional depiction of selected contacting amino acid residues of the binding pocket rendered in line and colored according to cartoon. Binding interaction residues are illustrated in yellow. Heteroatom color-code: V: grey, N: blue, and O: red. Molecular docking simulations of both ligands were performed individually. Hydrogen atoms are omitted for clarity. The final structure was ray-traced and illustrated with the aid of PyMol Molecular Graphics.

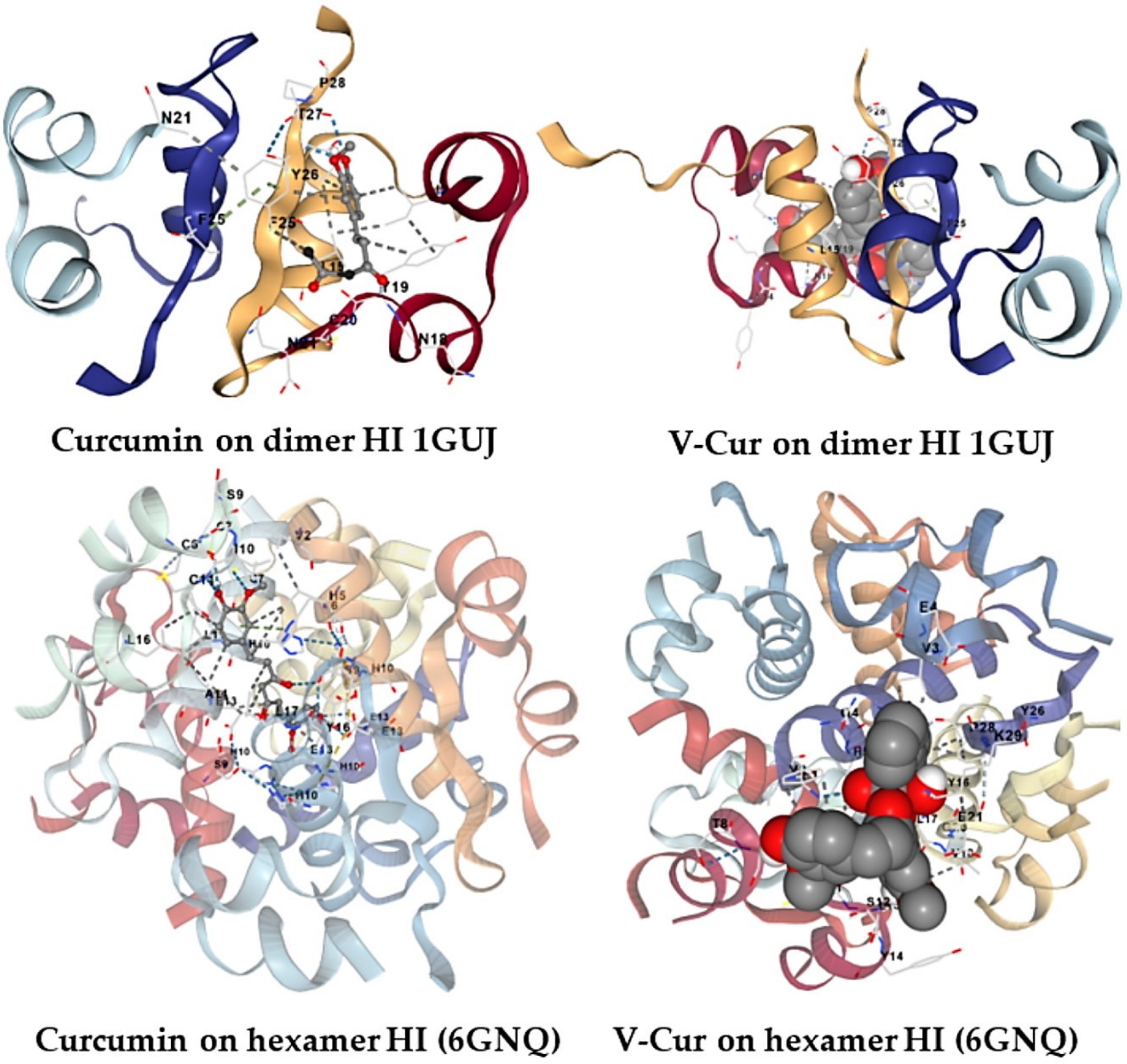

Figure S7. Binding interaction architecture of curcumin and V-Cur in the ligand-binding site of dimer (PDB ID 1GUJ) and hexamer (PDB ID 6GNQ) HI target proteins.

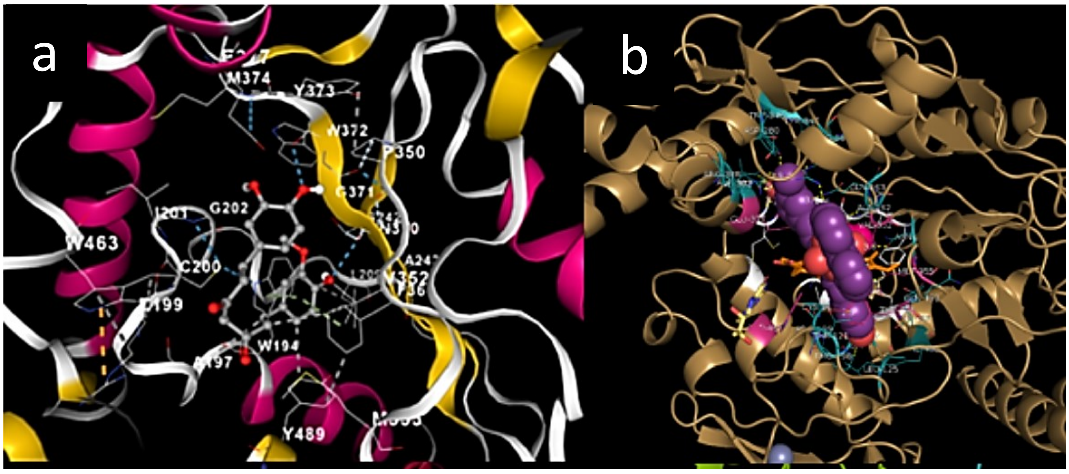

Figure S8. Binding interaction architecture of curcumin and V-Cur in the ligand-binding site of iNOS (a and b, respectively)
